# Clinical Performance and Implementation of AI-Enabled Paediatric Ophthalmic Screening, Triage, Diagnosis, and Surveillance in Primary, Community, and Referral-Linked Pathways: A Systematic Review

**DOI:** 10.3390/diagnostics16152389

**Published:** 2026-07-29

**Authors:** Joel Somerville, Mohammad Hussein Mustafa, Mohamed Mahmoud Seweid, Rabie Adel El Arab

**Affiliations:** 1 Centre for Rural Health Science, University of the Highlands and Islands, Inverness IV2 3JH, UK; joel.somerville@uhi.ac.uk; 2 Dr. Sulaiman Alhabib Medical Group, King Fahad Road, Olaya, Riyadh 11643, Saudi Arabia; 3 Faculty of Nursing, Beni-Suef University, Beni-Suef, Egypt; 4 Department of Nursing, Mohammed Al-Mana College for Medical Sciences, Dammam 34222, Saudi Arabia

**Keywords:** artificial intelligence, machine learning, paediatric ophthalmology, ophthalmic diagnostics, vision screening, retinopathy of prematurity, retinoblastoma, amblyopia, diagnostic accuracy, human-in-the-loop AI

## Abstract

**Background/Objectives**: Artificial intelligence (AI) is increasingly being evaluated for ophthalmic diagnosis, screening, and triage, yet its role in paediatric eye care remains less established than in adult ophthalmology. This systematic review aimed to synthesise evidence on AI-enabled tools for paediatric ophthalmic diagnosis, screening, triage, surveillance, and referral, with an emphasis on diagnostic performance, safety, workflow integration, equity, and implementation readiness in primary, community, and primary care-relevant settings. **Methods**: A PRISMA-guided systematic review was conducted using MEDLINE, Embase, Web of Science, Scopus, and IEEE Xplore from inception to 30 March 2026. Eligible studies evaluated AI or machine-learning tools for children and adolescents aged 0–18 years in relation to paediatric eye conditions. Study selection and data extraction were undertaken independently by reviewers, with disagreements resolved by consensus or third-reviewer adjudication. Methodological and reporting quality was evaluated using an author-adapted six-domain rubric informed by APPRAISE-AI. Diagnostic-accuracy studies were assessed using an author-adapted QUADAS-2 framework incorporating QUADAS-AI-informed AI-specific considerations, the prediction-model study was assessed using PROBAST+AI, and the non-randomised treatment-effect study was assessed using ROBINS-I. The public dataset descriptor was evaluated separately using an author-developed dataset-quality, representativeness, and applicability framework. Because of clinical and methodological heterogeneity, findings were synthesised thematically. **Results**: Twelve empirical studies and one public dataset descriptor were included, covering retinopathy of prematurity, retinoblastoma, amblyopia risk, myopia, congenital cataract, and visual-acuity assessment. AI systems frequently demonstrated promising diagnostic or screening performance, including sensitivity-first detection of treatment-requiring retinopathy of prematurity, high discrimination for retinoblastoma activity, and strong myopia prediction using fundus images. Several studies supported feasibility in neonatal, school, and community workflows using smartphone-based imaging, task-shifted operators, tele-referral, and human-in-the-loop review. However, external and temporal validation, calibration, patient-level reporting, subgroup and fairness assessment, and economic evaluation were limited. **Conclusions**: AI-enabled tools show promise for supporting selected paediatric ophthalmic screening, triage, and surveillance pathways, particularly when combined with image-quality control, explicit escalation, and human oversight. However, confidence in the reported performance is limited by single-centre studies and enriched samples, small numbers of clinically important cases, heterogeneous analytical units, potentially optimistic aggregation procedures, limited external or temporal validation, incomplete calibration, and absent fairness analyses. Routine autonomous implementation remains premature.

## 1. Introduction

Childhood vision underpins learning, social participation, and lifelong opportunity. Recent evidence links paediatric visual impairment to poorer literacy, educational attainment, and reduced quality of life, underscoring the importance of early detection and treatment [1,2]. Blindness is also strongly associated with poverty because it reduces economic productivity; in turn, poverty is a determinant of a wide range of health outcomes [3,4]. The importance of primary eye-care professionals in preventing, detecting, and treating conditions that can impair vision is recognised through their inclusion in public health systems [5,6].

Paediatric primary eye care is particularly important because visual development is experience-dependent and time-sensitive. During infancy and early childhood, coordinated visual input from the two eyes supports the maturation of binocular function and cortical visual pathways. Disruption of normal visual experience by strabismus, anisometropia, media opacity, or other forms of visual deprivation during sensitive developmental periods can lead to amblyopia and persistent visual deficits; early detection and appropriate treatment are therefore essential [7,8]. Optometrists are ideally placed within the health system to prevent amblyopia, detect its early signs, and provide timely interventions.

Several sight-threatening ocular conditions arise during infancy or childhood and may cause lifelong visual impairment when detection or treatment is delayed. These include retinopathy of prematurity in preterm infants, retinoblastoma, progressive myopia and other refractive disorders, congenital or infantile cataract, and primary congenital or juvenile glaucoma. Early recognition is essential because retinopathy of prematurity and retinoblastoma may progress rapidly, while cataract, glaucoma, and significant refractive error can disrupt visual development or cause irreversible ocular damage [9,10,11].

Retinopathy of prematurity (ROP) in particular persists as a major, avoidable cause of childhood vision loss, with contemporary analyses showing substantial burden across regions and rising screening demands as neonatal survival improves [12,13]. Retinoblastoma—though rare—exhibits stark, income-linked survival gaps, and delays in detection and referral carry irreversible costs [14]. Because delayed recognition of these conditions can result in irreversible visual impairment—and, in the case of retinoblastoma, may threaten survival—prompt detection and appropriate referral are essential components of paediatric primary eye care [8,15].

Meeting the need to detect, prevent, and treat childhood eye conditions through traditional models is increasingly difficult. Workforce projections indicate a widening mismatch between primary eye care supply and demand, with pronounced rural shortfalls [16]. Primary eye-care professionals also need to be appropriately distributed and equipped with sufficient instrumentation to diagnose and monitor eye conditions [16].

Paediatric eye care often depends on effective coordination between community-based eye-care professionals and specialist ophthalmology services. Collaborative-care programmes highlight the importance of clear referral pathways, shared clinical protocols, effective communication, professional education, and strong relationships between optometrists and ophthalmologists [17]. Access to eye examinations for young children may nevertheless be constrained by insufficient appointment time, a lack of age-appropriate equipment, gaps in paediatric training and clinical skills, and variable practitioner confidence, as documented in community optometric practice in England [18].

More broadly, substantial international disparities in the availability, distribution, training, and regulation of the optometric workforce may restrict equitable access to primary eye care, including services for children, particularly in lower-resource settings [19,20].

These trends threaten equitable access to paediatric eye care in primary and community settings [21]. Artificial intelligence (AI) is one intervention that could play a role in overcoming some of these challenges and improving the detection, monitoring and treatment of paediatric eye conditions.

Paediatric care also poses challenges, including infant motion, variable cooperation, and heterogeneous capture hardware, which demand sensitivity-first operation, robust uncertainty handling, and clear escalation rules if AI is to be used safely outside specialist centres [22,23,24].

Artificial intelligence, including machine learning and deep learning, is becoming an increasingly important tool in eye care [25]. Studies have demonstrated substantial advances in the application of AI to common adult eye conditions, including the detection and grading of diabetic retinopathy [26], the identification of glaucomatous changes, prediction of disease progression [27], and the detection and classification of age-related macular degeneration [28]. AI techniques used in adult eye care include the automated analysis of fundus photographs, optical coherence tomography scans, and visual-field data, as well as algorithms for disease classification, risk stratification, and prediction of disease progression [29].

The paediatric evidence remains fragmented and operationally under-characterised. Prior work focuses either on adult disease, individual paediatric indications—most often ROP—or tertiary workflows, leaving key questions for first-contact care unanswered—namely, how AI performs when operated by non-specialists; how image-quality control, escalation of uncertainty, and human–AI teaming are implemented; and whether models transport across devices, sites, and populations at acceptable safety and equity thresholds [30,31]. Methodological evaluations of clinical AI also flag recurring deficits—limited external/temporal validation, weak calibration reporting, and sparse subgroup and fairness analyses [32,33], all of which complicate adoption in community programmes serving diverse children.

A further barrier to equitable scale is the paucity of open, paediatric-relevant datasets with transparent labelling and recommended patient-level splits to prevent leakage. New resources are emerging; for example, an openly released, expert-labelled posterior-pole ROP image bank intended for screening/triage benchmarking—but these are still rare relative to clinical need. The health-system value case for paediatric AI is also only partially built beyond headline AUCs; decision-grade evidence on throughput, task-shifting, referral impact, and costs in real service pathways is limited, even as global commissions urge digital solutions that extend primary eye care [30,34,35,36].

Despite accelerating innovation, the evidence base for artificial intelligence in paediatric eye care remains fragmented and poorly aligned with the realities of first-contact services.

### 1.1. Aim

This review aimed to synthesise and critically appraise the performance, feasibility, safety, and health-system value of AI-enabled tools for paediatric eye care in primary/community settings and in primary care-relevant studies conducted in specialist environments, translating the evidence into guidance for equitable, scalable adoption.

### 1.2. Objectives

1. To quantify task-specific diagnostic and prognostic performance across paediatric eye conditions, benchmarking AI against clinicians, devices, and human–AI teams across settings;
2. To evaluate real-world feasibility and safety of deployment in primary/community pathways, including task-shifting, acquisition quality control, explainability, and escalation/uncertainty handling;
3. To assess transportability, equity, and health-system value across devices, sites, and populations, and to evaluate the role of open datasets and cost envelopes in scalable adoption.

## 2. Methods

### 2.1. Study Design

We conducted a systematic review reported in accordance with the PRISMA 2020 statement [37], using a thematic synthesis [38,39,40] to interpret heterogeneous evidence on AI-enabled tools for paediatric eye care among children and adolescents aged 0–18 years across screening, triage, surveillance, diagnosis, prognostication, and treatment evaluation in primary or primary care-relevant contexts. Conceptual framing used PICo (Population–Interest–Context) (Table 1) because the questions focused on the index test and context rather than on exposure–outcome relationships [41]; SPIDER (Table 2) was employed in parallel to structure feasibility, safety architecture, throughput, quality control, escalation logic, and other operational outcomes that informed the thematic analysis [41]. The review was not prospectively registered in PROSPERO or another systematic-review registry. The eligibility criteria, information sources, search strategy, study-selection process, data-extraction framework, synthesis approach, and appraisal methods are reported in full in the Methods and Supplementary Materials.

SPIDER is used to structure non-accuracy evidence for the thematic synthesis (feasibility, safety architecture, workflow, equity/scale/economics), complementing PICo’s focus on the index test and context in screening and diagnostic questions.

We defined primary care-relevant studies as those not conducted in primary or community settings but whose AI tool, dataset, or workflow was explicitly designed to support screening, triage, or surveillance hand-offs to primary/community care (e.g., school screening, tele-ophthalmology referral from non-specialists, neonatal screening), or whose outputs map directly to primary-care actions (refer/review/monitor).

### 2.2. Eligibility Criteria

The inclusion and exclusion criteria applied in the review are presented in Table 3.

### 2.3. Information Sources and Search Strategy

We designed a database-specific strategy to capture evaluations of AI-enabled tools for paediatric eye care across screening, triage, surveillance, diagnosis, prognostication, and deployment in primary or primary care-relevant contexts. We combined controlled vocabulary (MeSH/Emtree) and text words for four concept blocks: paediatrics, ophthalmic conditions, artificial intelligence/machine learning, and primary/community care pathways (including tele-ophthalmology, photoscreening, task-shifting, and quality control). Strategies were adapted to each platform’s syntax and field tags. We searched MEDLINE (PubMed), Embase (Ovid), Web of Science Core Collection, Scopus, and IEEE Xplore from inception to 30 March 2026. Full reproducible strings for every database are provided in Supplementary Materials (S1).

### 2.4. Selection Process

The records identified through database searches were imported into Rayyan, a systematic review screening tool [42]. Two independent reviewers screened the titles and abstracts against the inclusion and exclusion criteria. Full-text articles were subsequently retrieved for studies deemed potentially relevant. Any discrepancies between reviewers were resolved through discussion or consultation with a third reviewer to reach consensus.

### 2.5. Data Extraction and Synthesis

Two reviewers independently extracted data from all included studies using a predefined extraction framework. Extracted data included country and setting, care context, target condition, population and sample characteristics, AI tool or model type, input data, comparator, diagnostic or prognostic performance, deployment model, workflow relevance, key findings, and limitations affecting transferability. Disagreements were resolved through discussion, and unresolved conflicts were adjudicated by a third reviewer.

The extracted evidence was organised in Table 4 and synthesised thematically. Synthesis was anchored to the PICo framework, with SPIDER used to structure feasibility and operational data, including sensitivity-first thresholds, refer-on-inconclusive rules, human–AI teaming, quality-control yield, throughput, hardware or operator dependencies, and calibration reporting. Findings are presented at the patient level where possible, with eye- or image-level results clearly flagged. No statistical pooling was attempted because of heterogeneity in thresholds, units of analysis, settings, operators, and hardware; when multiple thresholds were reported, the operational or prespecified sensitivity-first threshold was prioritised.

For each study, we additionally recorded the model-input unit, data-partitioning unit, performance-reporting unit, aggregation procedure, inclusion of both eyes, and handling of within-child correlation. These features were used to determine whether frame-, image-, eye-, examination-, and patient-level results were directly comparable and to assess the potential influence of aggregation and clustering on reported performance.

### 2.6. Quality Assessment Methods

Methodological and reporting quality was assessed separately from risk of bias. For the 11 studies that developed or evaluated AI models for clinical decision support, two reviewers independently applied an author-adapted six-domain quality rubric informed by APPRAISE-AI [43]. The domains were clinical relevance, data quality, methodological conduct, robustness and generalisability, reporting quality, and reproducibility and transparency.

The adaptation retained the broad conceptual domains of APPRAISE-AI but did not represent an unmodified application of the original 24-item weighted instrument. Each domain was scored from 0, representing the lowest level of methodological or reporting quality, to 5, representing the highest level. The six equally weighted domain scores were summed to produce a descriptive total ranging from 0 to 30.

Total scores were not converted into categorical ratings such as Excellent, Good, Moderate, or Poor. A summed score could conceal important weaknesses because stronger performance in clinical relevance or reporting could compensate numerically for limitations in external validation, calibration, representativeness, analytical-unit selection, clustering, transparency, or reproducibility. Domain-level findings were therefore interpreted descriptively rather than as measures of evidence of certainty or implementation readiness.

The assessment also recorded AI-specific methodological concerns, including separation of model-development and evaluation data at the patient level, potential information leakage, threshold selection, unit of analysis, aggregation across frames, images, or eyes, within-child clustering, calibration, independent external or temporal validation, model transparency, and availability of code, data, or other reproducibility artefacts.

The FARFUM-RoP dataset descriptor was assessed separately using the author-developed dataset-quality, representativeness, and applicability framework. The non-randomised treatment-effect study by Han et al. was evaluated principally using ROBINS-I [44], because its primary objective was estimation of treatment effectiveness using an ML-derived counterfactual rather than development or validation of an AI clinical decision-support model.

The two reviewers reconciled differences through discussion and reached consensus on the final domain scores. Where consensus could not be reached, the assessment was adjudicated by a third reviewer. Quality-rubric findings were interpreted alongside the design-specific risk-of-bias assessments, which were given greater weight than the summed scores when determining the strength of the synthesis and conclusions.

### 2.7. Risk of Bias Assessment

We used a design-aligned, AI-aware strategy spanning four evidence streams: diagnostic accuracy studies (QUADAS-AI) [45] were assessed using an author-adapted QUADAS-2 framework [46], prediction model studies (PROBAST + AI) [47], non-randomised treatment effectiveness studies (ROBINS-I) [44], and dataset descriptors, which were evaluated separately using an author-developed dataset-quality, representativeness, and applicability framework. This approach matches each study’s causal target with the domains most likely to harbour bias, while explicitly capturing AI-specific threats (e.g., data leakage, calibration deficits, and fairness).

Tool selection and justification. QUADAS-AI for AI diagnostics, interrogating patient selection, index test, reference standard, and flow/timing while adding probes for separation of training/tuning/testing at the patient level, threshold pre-specification, calibration of probabilistic outputs, model transparency/versioning, and subgroup/fairness performance. PROBAST + AI appraises prediction studies across participants, predictors, outcome, and analysis, with ML-specific checks for leakage, nested internal validation during tuning, handling of missingness and class imbalance, clustering (e.g., both-eyes data), external/temporal validation, calibration, and threshold derivation. ROBINS-I is the accepted standard for interventional questions without randomization; we treat ML-based pseudo-controls as potential sources of outcome measurement and confounding bias (through prediction error/miscalibration and overlap with treatment assignment). For datasets, we applied an author-developed descriptive framework with the following domains: Selection and Representativeness; Labelling/Reference Standard; Filtering/Exclusions; Provenance and Versioning; Preprocessing Transparency; Annotation Reliability and Uncertainty; Bias and Fairness; Documentation and Accessibility; Overall Dataset Concern; Key Concerns/Flags; and Next Actions/Clarifications. These domains were adapted from established data governance and transparency frameworks, including Datasheets for Datasets [48], Model Cards for Model Reporting [49], and recommendations for bias assessment in AI healthcare reporting from CONSORT-AI [50]. Together, these sources provided a structured basis to capture representativeness, labelling quality, documentation, and fairness—elements not comprehensively addressed by existing study-level RoB tools.

Operationalization. For each tool, we developed anchor criteria for Low (L), Some concerns (S), and High (H) (ROBINS-I uses its native grades). QUADAS-AI “High” included any critical flaw such as leakage, incorporation bias between index and reference, opaque or post hoc thresholds, absent calibration, or unmasked/weak reference standards. PROBAST + AI “High” included invalid analysis pipelines (e.g., tuning on the test set), lack of external/temporal validation when claimed for deployment, ignored clustering, or missing calibration. ROBINS-I judgments prioritised confounding and outcome measurement; unmeasured confounding, cycloplegic/manifest mismatches, or failure to account for clustering elevated the overall grade. For datasets, a high overall level of concern was assigned when any foundational domain—Selection and Representativeness, Labelling/Reference Standard, or Provenance and Versioning—was High, or when ≥3 domains were High.

Two reviewers independently assessed each study using the applicable design-specific tool or dataset framework. Differences in domain-level judgements were resolved through discussion, with unresolved disagreements adjudicated by a third reviewer. Final consensus judgments and the principal reasons supporting each judgment are reported in Supplementary Materials (S3). No formal inter-rater agreement statistic was calculated.

## 3. Results

### 3.1. Study Selection

**Figure 1:**
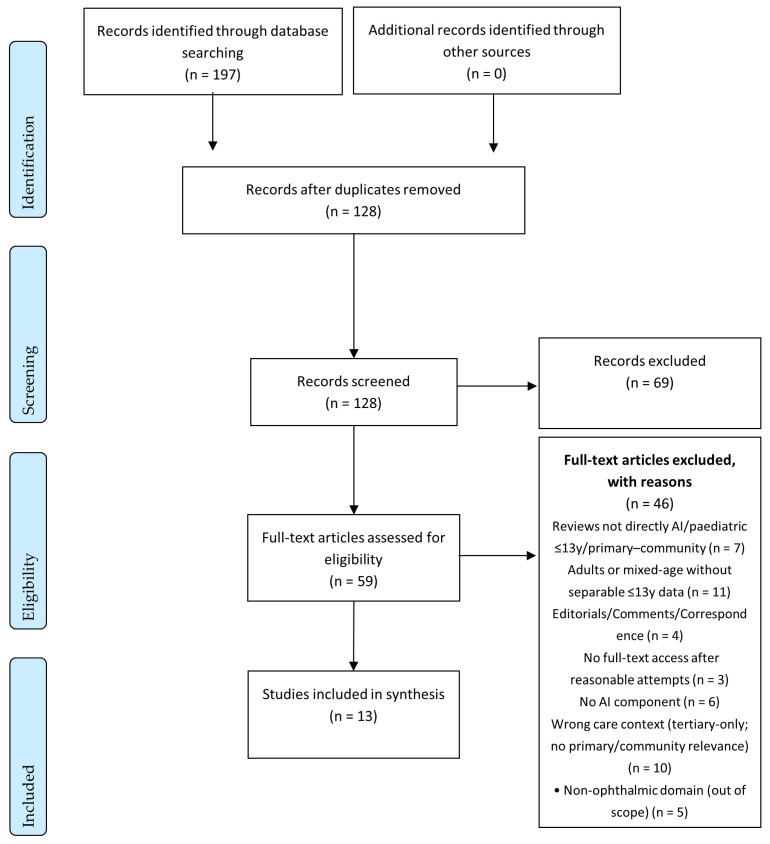
PRISMA flow diagram of study identification, screening, eligibility assessment, and inclusion.

The database search identified 197 records, and no additional records were identified through other sources. After duplicate removal, 128 records remained for title and abstract screening. Of these, 69 records were excluded, and 59 full-text articles were assessed for eligibility. Forty-six full-text articles were excluded for the following reasons: reviews not directly focused on AI in paediatric populations aged ≤18 years or not relevant to primary/community care (*n* = 7); adult-only or mixed-age studies without separately extractable data for participants aged ≤18 years (*n* = 11); editorials, comments, or correspondence articles (*n* = 4); no full-text access after reasonable attempts (*n* = 3); no AI component (*n* = 6); wrong care context, such as tertiary-only studies without primary/community relevance (*n* = 10); and non-ophthalmic domain studies (*n* = 5). Thirteen records met the eligibility criteria and were included in the review: 12 empirical studies and one public dataset descriptor. The empirical evidence comprised 11 AI model-development or validation studies and one non-randomised treatment-effect study using an ML-derived counterfactual (Figure 1).

### 3.2. Characteristics of Included Studies

The review included 12 empirical studies and one public dataset descriptor. Eleven empirical studies developed or evaluated AI models for diagnosis, screening, triage, surveillance, or prediction, while one non-randomised study used an ML-derived natural-history model to estimate treatment effectiveness. The public FARFUM-RoP report described a dataset intended to support AI development and benchmarking and was analysed separately from the clinical model studies [51,52,53,54,55,56,57,58,59,60,61,62,63]. The included evidence originated from China, India, Singapore, Spain, Hungary, the USA, Mexico, Argentina, Iran, and Korea. The target conditions and clinical functions included ROP screening, retinoblastoma surveillance, amblyopia and amblyogenic-risk detection, congenital-cataract identification, myopia detection and long-term risk prediction, early-childhood visual-acuity assessment, and treatment-effect estimation.

Model inputs varied substantially across studies. They included wide-field and smartphone-based fundus photographs, smartphone fundus videos, external ocular-appearance images, visual-acuity examination videos, tablet-based stereovision tests, photoscreener outputs, and structured clinical or perinatal variables. The evaluated methods included convolutional neural networks, residual and DenseNet architectures, transfer learning, frame-selection models, perceptron-based composite classifiers, random forests, AdaBoost, human-in-the-loop pipelines, and combined image-plus-clinical prediction models.

The studies also represented different stages of clinical and operational maturity. Evaluations were conducted in specialist clinics, neonatal units, school-screening programmes, tele-ophthalmology services, and preliminary rural or resource-constrained workflows. Several studies assessed acquisition by nurses, technicians, teachers, or other non-specialist operators, whereas others remained dependent on specialist-centre imaging, examination under anaesthesia, proprietary systems, or research infrastructure. Complete study-level characteristics, performance estimates, deployment settings, and limitations are presented in Table 4.

**Table 4:** Characteristics of included studies.

Author(s)-Year	Country/Setting	Target Condition(s)	Population/Sample	AI Tool Type/Model	Data Input(s)	Diagnostic Performance	Deployment/Setting	Key Findings	Limitations
Han et al. 2025 [51]	Korea; Kim’s Eye Hospital, Seoul; Evidence type: Non-randomised treatment-effect evaluation using an ML-derived counterfactual.	Childhood myopia (0.125% atropine).	771 eyes (397 children); 6–30 mo groups.	ML natural-course predictor (56,933 refs; RMSE 0.817 D).	Baseline cycloplegic SE, age/sex, spherical/cylindrical errors, Δage.	Average suppression was approximately 53.5%; effects were significant at ≥12 months.	Single-centre, ML as simulated control.	The ML-counterfactual analysis estimated lower progression during atropine treatment, although causal confidence was limited by serious risk of bias; fewer non-responders with longer duration.	Single site; nonrandomized; both eyes included; ML prediction error; generalizability.
Zhang et al. 2023 [52]	China; Beijing Tongren Hospital; single-centre.	Retinoblastoma (normal vs. stable vs. active)	Dev: 36,623 images (713 pts); Prospective: 103 pts, 139 eyes.	ResNet-50 (two binary classifiers).	RetCam fundus images acquired during examination under anaesthesia, including the posterior pole and 12 peripheral clock-hour views.	Prospective AUC 0.991 (normal–active), 0.962 (stable–active); sens 0.979, spec 1.000.	EUA workflow at tertiary eye centre; telemedicine-ready, cost-effective.	Demonstrated high discrimination and improved reader performance in a single-centre prospective evaluation; the economic findings require confirmation in independent health systems.	Single-centre; image-quality control needed; operator-dependent; small prospective sample (103 pts).
Luo et al. 2023 [53]	China; remote/rural tele-ROP via edge–cloud telemedicine, deployed with support of a core Guangdong provincial hospital.	Retinopathy of prematurity.	900 fundus images (500 ROP, 400 normal) from Guangdong Maternal and Child Health Hospital; expert grading, augmented.	ResNet101-based classifier with undersampling/resampling and label-distribution margin loss in an edge–cloud collaborative architecture.	Manually labelled colour fundus images (privacy-stripped, resized to 224 × 224), augmented and preprocessed.	Test performance: ~75% accuracy, 60% AUC.	Embedded in an Android mobile app with edge–cloud screening workflow for remote ROP detection.	Edge–cloud DL system partially mitigates class imbalance and enables remote screening; moderate diagnostic accuracy with potential to expand access.	The small, imbalanced dataset limited accuracy; larger datasets and further optimisation are needed.
Foo et al. 2023 [54]	Singapore; school-based SCORM cohort with internal (Schools 2&3) and external (School 1) validation.	5-year risk of high myopia (SE ≤ −6.00 D).	998 children (1878 eyes), 7456 baseline fundus images; external validation 99 children (189 eyes).	Deep learning system: image-only (DenseNet-121), clinical (random forest), and mixed image + clinical models.	Baseline fundus photographs; clinical variables (baseline SE, age, race and gender); optional 1-year SE progression.	Mixed model AUC 0.97 internal and 0.97–0.98 external; fundus-only AUC ~0.93 both; baseline SE model AUC 0.90 (internal), 0.93 (external).	School-based screening/prediction with potential for community/school deployment.	Mixed model performed best; fundus-only nearly comparable; minimal incremental gain from 1-year progression.	Singapore-only cohort with imbalanced high myopia distribution; needs broader external validation; reliance on cycloplegic mydriatic imaging; limited axial length prediction due to small sample.
Akbari et al. 2024 [55]	Iran; ROP Ward, Farabi Eye Hospital (Tehran); Evidence type: Public dataset descriptor; no AI model performance evaluated.	Retinopathy of Prematurity/Plus Disease.	68 preterm infants (GA < 34 weeks, BW < 2000 g), 1533 fundus images labelled by 5 ophthalmologists.	N/A (public dataset for AI development)	Wide-angle posterior retinal fundus images (RetCam) after pupil dilation; expert quality filtering.	N/A; expert consensus grading (Normal, Pre-Plus, Plus).	Public FARFUM-RoP dataset on Figshare, collected in-clinic	Publicly accessible exploratory ROP image resource with multi-reader annotations; useful for model development but not a deployment-grade benchmark.	Single-centre origin; removal of low-quality images; image-level rather than examination-level labelling; no reported formal inter-rater reliability statistic; no recommended patient-level development/test partitions.
Ortiz et al. 2025 [56]	Mexico & Argentina; NICUs in low-resource settings using smartphone video with low-cost magnifier.	Retinopathy of prematurity screening.	512 preterm neonates (524 videos; GA < 36 weeks or BW < 1500 g).	Frame-selection CNN + 18-layer ResNet classifier (sensitivity-optimised with temperature scaling).	Smartphone-captured fundus video frames, best-quality retinal images selected.	Frame-level sensitivity 76.7%; patient-level sensitivity 93.3%; higher sensitivity than ophthalmologists but lower specificity/accuracy.	Point-of-care smartphone-based screening with minimal training, immediate upload/evaluation for quality control in low-resource NICUs.	High sensitivity enabling effective ROP case detection and potential to expand screening access in resource-limited settings.	Limited generalizability (Latin America only), comparison against only 3 ophthalmologists, lower specificity/accuracy, variability in image quality.
Arnold et al. 2022 [57]	USA; paediatric ophthalmology clinic (Alaska Blind Child Discovery, Anchorage, AK).	Amblyopia risk factors (refractive + strabismus per AAPOS).	200 children aged 0.4–18 years, ethnically diverse, ~35% with developmental delays.	AI Optic (central AI interpretation) vs. Plusoptix a-12 (local infrared photoscreener with in-device interpretation).	Multi-radial infrared photoscreen images for refractive (sphero-cylinder) and strabismus assessment with ROC-based amblyopia risk factor screening.	≥4y targeting 2021 refractive + strabismus ARF: AI Optic AUC 0.58 vs. Plusoptix 0.74; refractive estimation inferior by ABCD ellipsoid (*p* < 0.001).	Clinic-based handheld screening; AI Optic needs WiFi for central reading while Plusoptix works offline with real-time in-device interpretation.	Plusoptix outperforms AI Optic in amblyopia risk factor screening and refractive accuracy; AI Optic is lower cost and may improve with further validation.	Not community-based, small preschool subgroup, highly experienced screener (limits generalizability), and no formal cost–benefit analysis.
Ma et al. 2020 [58]	China; school-based vision screening in Luoyang, Henan (resource-limited primary schools).	Strabismus, myopia, anisometropia.	100 children aged 8–10 years (20 with suspected strabismus, 80 randomly selected).	Deep learning + image-processing pipeline combining automated Hirschberg test and photorefraction; facial/eye localization (e.g., landmark estimation) to derive risk.	Smartphone video/images capturing corneal luminous reflection and red reflex, QR code–based scale, head/eye pose from landmarks.	Strabismus: sens 0.80, spec 0.98; Myopia: sens 0.83, spec 1.00; Anisometropia: sens 0.80, spec 1.00.	Dark-room streamlined school workflow with one GP + teacher screening ~200 kids/hour using mainstream smartphones (iPhone 5s, Honor 8, Mi 6).	Demonstrated high throughput and promising screening performance in a preliminary evaluation conducted in two schools; minimal training needed.	Suboptimal flash control reducing signal-to-noise; non-generalised limbus detection (contrast-dependent); limited evaluation (only 2 urban schools, Chinese sample).
Lin et al. 2020 [59]	China; Zhongshan Ophthalmic Center national referral paediatric ophthalmology centre; online preclinical prediction platform.	Congenital cataracts (bilateral and unilateral).	2005 children (1274 CC cases, 731 healthy controls); training: 1129 CC vs. 609 controls; external validation: 145 CC vs. 122 controls.	Random Forest and Adaptive Boosting on imputed structured non-imaging data.	Birth conditions, family medical history, and environmental factors (11 non-imaging predictors).	High discrimination: internal CV bilateral RF AUC 0.91 (95% CI 0.88–0.94); external validation bilateral AUC ~0.93 ± 0.05; stable clinical-test AUC 0.94–0.96.	Tertiary centre case–control with internal, external and simulated low-prevalence clinical tests; web-based tool for broader testing.	Non-imaging model accurately identifies high-risk infants; top predictors: family history, low parental education, comorbidity; potential complementary screening in underserved areas.	Limited real-world external validation and potential selection bias; predominantly Chinese sample limits generalizability; possible misclassification (e.g., comorbidity reporting), age mismatch.
Young et al. 2023 [60]	India; single-centre tele-ROP telemedicine programme at Aravind Eye Hospital, Coimbatore.	Referral-warranted and treatment-requiring ROP (severe ROP).	156 premature infants (312 eyes) imaged Jan 2021–Apr 2022 by technicians.	ResNet18 deep learning binary classifier (preplus/plus); plus human grader vascular severity scoring.	Smartphone fundus images (MII Retcam & Keeler MIO) with WDFI reference.	Human graders: VSS AUC 0.95 (RW), 0.96 (TR); TR-ROP sensitivity 100% (spec ~83%); AI: TR-ROP sens 100% spec 58.6%, RW-ROP sens 80% spec 59.3%.	Technician-acquired smartphone imaging in operational telemedicine screening.	Smartphone-based imaging with human and AI grading achieved high sensitivity for TR-ROP under the selected operating threshold, although specificity was lower, and external validation is needed.	Small TR-ROP sample (n = 14); single-country (India) limits generalizability; limited peripheral/mild ROP detection; variability in acquisition/training; needs external validation.
Csizek et al. 2023 [61]	Spain & Hungary; paediatric ophthalmology screening at tertiary eye departments (Alicante & Pécs).	Amblyopia and amblyogenic conditions (strabismus, anisometropia, hyperopia).	423 children aged 3.6–14 years (amblyopia *n* = 46; amblyogenic *n* = 55; non-amblyogenic *n* = 128; emmetropic controls *n* = 194).	Perceptron-based AI-ETS combining four non-stereoacuity EuvisionTab stereograms with optimised weighting (linear integrator, logistic sigmoid + training via Levenberg–Marquardt).	Low-density static/dynamic anaglyphic random dot stereograms (ETS variants: SRDS 8, DRDS 1, DRDS 0.7, noise) aggregated into composite scores; tested with red-green goggles.	Near-perfect AUC for amblyopia (AI-aw NC 0.996; AI-aw WC 0.976) with 100% sensitivity at optimum point and superior detection of amblyopia/amblyogenic conditions versus classic tests.	Tablet-based ETS in dark room, administered by experienced clinicians at two sites; AI decision engine implemented in MATLAB (2018b) with weight optimisation.	AI-ETS (especially AI-aw) outperforms traditional stereovision tests; high sensitivity and AUC; practical for mass/community screening.	Incomplete systematic testing of all subjects, limited newly diagnosed cases, and wide age range beyond standard screening target (3.5–6 years).
Yang et al. 2020 [62]	China; Zhongshan Ophthalmic Center paediatric myopia AI programme, prospective clinical trial in schoolchildren.	Myopia (SER ≤ −0.5 D).	2350 ocular-appearance images from children aged 6–18 years; trial: 50 students (100 eyes).	VGG-Face deep CNN with transfer learning (DLS).	Three-angle stitched ocular appearance images (224 × 224) from various cameras of children.	Internal: AUC 0.9270 (81.13% sens, 86.42% spec); Clinical trial: AUC 0.9140, sens 84%, spec 74%, outperforming ophthalmologists.	Clinic-based prospective trial; uses regular camera/smartphone images enabling remote/home screening.	High accuracy; better than ophthalmologists; stable AUC across age/sex.	Variable sens/spec across subgroups; not validated in multiethnic populations; no quantitative diopter output.
Zhang et al. 2020 [63]	China; Zhongshan Ophthalmic Center paediatric vision clinic + cloud DL platform.	Early-childhood visual acuity abnormality	1885 TVA exam videos (plus 50 external), expert labelled.	Human-in-the-loop DL (Faster-RCNN + CNN + rule modules) vs. end-to-end LRCN.	TVA video frames.	Human-in-loop: 75.54% vs. LRCN: ~49%; localization AP > 85%.	Clinic exam with Django-based cloud backend.	Human + AI > human or AI alone; interpretable.	Occlusion/small-object detection issues; needs more data for age generalizability and robustness.

Legend: AUC = area under the receiver operating characteristic curve; AI = artificial intelligence; CNN = convolutional neural network; CV = cross-validation; DL = deep learning; EUA = examination under anaesthesia; GA = gestational age; LRCN = long short-term recurrent convolutional network; ML = machine learning; NICU = neonatal intensive care unit; ROP = retinopathy of prematurity; RW = referral-warranted; SE = spherical equivalent; sens = sensitivity; spec = specificity; TR = treatment-requiring; TVA = test visual acuity; VSS = vascular severity score; WDFI = widefield digital fundus imaging.

### 3.3. Thematic Synthesis

Across thirteen studies spanning retinopathy of prematurity (ROP), retinoblastoma (RB), amblyopia risk, congenital cataract, myopia detection/prediction, paediatric visual acuity, and treatment evaluation, four cross-cutting themes emerged: clinical performance; deployment and workflow feasibility; safety, interpretability, and decision support; and equity, scale, and value.

#### 3.3.1. Theme 1: Clinical Performance Across Paediatric Eye-Care Tasks

##### Neonatal and Oncologic Retinal Disease (ROP, RB)

Across neonatal ROP screening, smartphone posterior-pole imaging showed high discrimination under the evaluated study conditions: human vascular-severity scoring achieved AUC 0.95–0.96, and an autonomous classifier attained 100% sensitivity for treatment-requiring ROP (TR-ROP)—a deliberately sensitivity-first operating point appropriate for screening [60]. However, at the operating point used by Young et al. [60], the AI’s specificity for treatment-requiring ROP was only 58.6%, whereas human graders achieved 83.5% specificity at the same sensitivity.

Building on this, a smartphone-video pipeline improved end-to-end detection by pairing frame selection with approximately 97% precision with a calibrated residual network, yielding patient-level sensitivity of 93.3%, compared with 73.3% for the paediatric-ophthalmologist consensus (trade-off: lower specificity to minimise misses), but the system’s specificity and overall accuracy were lower than those of the ophthalmologist panel, and only three ophthalmologists were used as comparators [56].

By contrast, an edge–cloud ROP prototype demonstrated full telemedicine feasibility but only modest accuracy (~60–75%) in a small, imbalanced dataset—underscoring that data sufficiency and class balance, not only architecture, constrain performance at early maturity [53].

For oncologic surveillance, a prospectively validated deep-learning assistant for RB (DLA-RB) achieved an AUC of 0.991 (sensitivity 0.979 [0.927–1.000], specificity 1.000 [1.000–1.000]) for normal vs active cases and 0.962 (sensitivity 0.978 [0.932–1.000], specificity 0.800 [0.556–1.000]) for stable vs active cases. Three of the four misclassified normal vs active cases were false positives. Performance for the stable vs active task was also excellent; crucially, reader–AI collaboration outperformed readers alone, supporting further evaluation of high-sensitivity, clinician-supervised surveillance workflows [52]. The trial-embedded economic analysis suggested potential cost-effectiveness within the single-centre study setting, although transferability remains uncertain [52].

##### Amblyopia Risk and Paediatric Visual Function

In head-to-head clinic testing, the validated on-device photoscreener (PlusoptiX) outperformed a low-cost, centrally interpreted AI-Optic for amblyopia-risk detection (AUC ≥ 0.72 vs. 0.53–0.58) and provided more accurate sphero-cylinders (lower ABCD ellipsoid), despite producing more “inconclusive” results—evidence that conservative fail-safes can preserve validity in real clinics [57]. The AI Optic device produced fewer ‘inconclusive’ results but had significantly lower AUC and refractive accuracy than the Plusoptix photoscreener; thus, convenience came at the cost of diagnostic performance.

A complementary line of evidence shows that combining simple stereovision stimuli with lightweight AI yields high discrimination within the evaluated cohort: a Perceptron-weighted ensemble of EuvisionTab tests achieved the highest AUCs and often near-perfect sensitivity, significantly surpassing classic stereovision tests with stable generalisability across 100 independent reinitialisations [61].

For younger children’s visual-acuity estimation, human-in-the-loop deep learning that embeds clinician heuristics outperformed a fully automated baseline and individual ophthalmologists (achieved approximately 0.76 accuracy, compared with 0.49 for the end-to-end model and 0.56, 0.54, and 0.48 for the three ophthalmologists), highlighting the impact of structured human–AI collaboration [63].

##### Myopia, Congenital Cataract, and Treatment Response

Using a single baseline fundus photo, a deep-learning system predicted 5-year high -myopia risk with AUC ≈ 0.93 (image-only) and ≈0.97 when combined with baseline refraction, matching clinical models while remaining more scalable for school screening [54]. High-myopia prevalence in the cohort was low, and calibration and clustering (both eyes per child) were not reported; therefore, the positive predictive value and generalisability remain uncertain.

Even more minimal inputs can suffice: an ocular-appearance classifier using consumer devices prospectively outperformed ophthalmologists for binary myopia detection (AUC ≈ 0.914; sensitivity 84% vs. 64%; specificity 74% vs. 53%) [62].

Where imaging is scarce, non-imaging ML using history and perinatal factors identified congenital cataract with high discrimination, remaining robust down to 1:10 case:control simulations; interpretability analyses consistently ranked family history, parental education, and comorbidity as the top predictors—potentially actionable cues for primary-care triage [59]. This was a case–control study using simulated prevalence; results may overestimate performance compared with a true screening population.

AI also enabled treatment-effect quantification without RCTs: an externally validated natural-history model estimated ≈50–60% suppression of refractive progression with 0.125% atropine beyond 12 months, with non-responder rates falling as therapy lengthened [51].

##### Influence of Analytical Unit and Aggregation on Performance Estimates

The included studies reported performance using heterogeneous analytical units including individual video frames, images, eyes, examinations, and children. These units are not directly interchangeable, and the reported performance must be interpreted in relation to the unit at which model outputs were generated and clinical decisions were made.

Patient-level aggregation may appropriately reflect the intended clinical action when the decision concerns referral of the child. However, combining classifications across multiple frames, images, or eyes provides several opportunities for a child to generate a positive result. Depending on the aggregation rule and decision threshold, this may increase patient-level sensitivity while reducing specificity and increasing false-positive referrals.

This effect was evident in the smartphone-video ROP study by Ortiz et al., in which sensitivity increased from 76.7% at the frame level to 93.3% at the patient level. Patient-level specificity was 55.2%, indicating that the higher sensitivity was accompanied by a substantial false-positive burden. The patient-level estimate should therefore be interpreted as the performance of the complete frame-selection, aggregation, and thresholding strategy rather than as an equivalent improvement in the classification of each individual frame [56].

Studies that included both eyes from the same child also required appropriate handling of within-child correlation. Measurements from fellow eyes are generally correlated and cannot be treated as fully independent observations without potentially overstating the effective sample size and underestimating variance and confidence interval widths. This concern was particularly relevant to studies reporting eye-level results from multiple children in whom both eyes contributed observations, including the high-myopia prediction and ML counterfactual treatment-effect studies [51,54].

Accordingly, patient-level sensitivity was interpreted alongside the underlying frame-, image-, or eye-level results, the specified aggregation rule, specificity, and likely referral burden. Greater interpretive weight was given to studies that separated model-development and evaluation data at the patient level, prespecified their aggregation procedures, and used analytical methods appropriate for clustered observations. Table 5 summarises the analytical units, aggregation procedures, dependence structures, and likely implications for the reliability of the reported estimates in studies where these issues materially affected interpretation.

#### 3.3.2. Theme 2: Deployment Models and Workflow Feasibility

##### Commodity Hardware and Cost Envelopes

In a prospective single-centre ROP screening study in India, technician-acquired smartphone fundus imaging combined with a ResNet18 classifier achieved 100% sensitivity for treatment-requiring ROP at the chosen operating point, with lower specificity [60].

Similarly, a one-step, high-throughput school screening workflow, executable by minimally trained staff, ran on outdated mainstream smartphones (iPhone 5s, Honor 8, Mi 6), using hybrid AI-enabled analysis, pose control, and scale calibration to achieve sensitivities ≥0.80 and near-perfect specificities while screening ~200 children/hour demonstrating the feasibility of smartphone-based screening and promising performance under the evaluated school conditions [58]. These throughput findings suggest operational efficiencies; however, no formal cost-effectiveness or budget-impact analysis was conducted, so the economic value of this workflow remains unknown.

##### Task-Shifting, Training, and Throughput

In a single-centre ROP screening programme in India, task-shifting was feasible: general nurses, after a structured 4-week training, captured smartphone fundus images of sufficient quality for both masked expert grading and ResNet18 AI analysis, enabling accurate detection of treatment-requiring ROP and supporting referral decisions [60].

In schools, a teacher and a supervising GP screened approximately 200 children per hour, with 87% of children completing the screen on the first attempt and 100% within two attempts—throughput levels that change population-scale feasibility [58].

In a three-step, smartphone-based neonatal ROP screening pipeline in Mexico and Argentina, automated frame selection achieved 97.4% precision in identifying clear fundus images from noisy video, reducing unnecessary recapture and providing standardised, high-quality inputs for downstream AI classification that prioritised sensitivity for screening [56].

##### Edge–Cloud and Tele-Ophthalmology Integration

In a preliminary ROP tele-screening system, a three-layer edge–cloud framework—local fundus image capture and preprocessing, cloud-based ResNet101 inference, and triage feedback—reduced bandwidth demands and concentrated hub specialist input on flagged cases. The Android mobile interface integrated image acquisition and cloud connectivity, enabling capture → AI → feedback loops at remote spoke sites, though current deployment remained cloud-dependent and model performance was modest (~60–75% accuracy/AUC) due to limited, imbalanced training data [53].

#### 3.3.3. Theme 3: Safety, Interpretability, and Decision Support

##### Sensitivity-First Operating Points and Escalation Logic

Both ROP systems used sensitivity-prioritised operating points that explicitly prioritised sensitivity over specificity to minimise the risk of missing severe disease. In the Indian SBFI study, the ResNet18 classifier was tuned to achieve 100% sensitivity for treatment-requiring ROP (with reduced specificity) [60], while in the neonatal video pipeline, an 18-layer residual network optimised for recall reached 93.3% patient-level sensitivity, outperforming ophthalmologist consensus, reflecting a deliberate acceptance of more false positives in exchange for safeguarding against false negatives [56].

In this head-to-head paediatric photoscreening study, treating inconclusive results as referrals preserved sensitivity. Even so, the established on-device PlusoptiX a-12, despite producing more inconclusive results, achieved higher validity for amblyopia risk factor detection than the lower-cost, WiFi-dependent AI Optic with centralised AI interpretation [57].

In a prospective validation across two RetCam3 generations, the DLA-RB system achieved near-perfect discrimination for normal vs. active retinoblastoma (AUC = 0.991; sensitivity = 0.979; specificity = 1.000) and excellent performance for the harder stable vs. active task (AUC = 0.962; sensitivity = 0.978; specificity = 0.800), suggesting potential to support clinician-supervised de-escalation of stable eyes while ensuring timely escalation of active disease for treatment [52].

##### Explainability Aligned with Pathology

Explainability outputs in ophthalmic AI are often co-localised with clinically meaningful anatomy. Grad-CAM in retinoblastoma detection [52] highlighted tumour and relapse loci; AI focus in ROP adjudication [60] corresponded to posterior-pole vasculature used in clinical grading; and Grad-CAM in ocular-appearance myopia detection [62] emphasised temporal sclera/periocular regions. Where applied, saliency mapping techniques such as Integrated Gradients in high-myopia risk modelling [54] identified optic disc and macular areas, supporting anatomical plausibility, although the effect of these explanations on clinician decisions or patient safety was not evaluated.

##### Human–AI Collaboration Compared with Autonomous Approaches

In evaluated high-stakes ophthalmic tasks, combined human–AI workflows showed higher performance than the selected comparators in two included studies: in retinoblastoma detection [52], reader + AI surpassed readers alone in both screening and surveillance; in paediatric acuity assessment [63], a human-in-the-loop system achieved 75.5% accuracy versus 49.0% for an end-to-end model and 48–56% for individual ophthalmologists. By contrast, in paediatric photoscreening [57], a low-cost, centralised “black-box” AI device (AI Optic) delivered faster outputs and fewer inconclusives than the validated on-device PlusoptiX, but with consistently lower AUCs and refractive estimation accuracy, underscoring that convenience cannot replace calibrated thresholds and conservative fail-safes in safety-critical screening. These comparative findings remain preliminary because they arose from a small number of single-centre studies and were not linked to patient-important outcomes.

#### 3.3.4. Theme 4: Equity and Scale

##### Open Datasets and Benchmarking

FARFUM-RoP provides a publicly accessible collection of 1533 posterior retinal images from 68 premature infants, with annotations supplied by five ophthalmologists. Its accessibility is valuable in a field in which many development datasets remain private [55].

Its suitability for downstream benchmarking is nevertheless constrained. The images originated from a single specialist centre, and approximately 30 low-quality images were removed. This curation reduces representation of poor focus, motion blur, low contrast, uneven illumination, incomplete fields, and other acquisition failures that an operational screening model would need to manage.

Labels were assigned to individual images, although clinical ROP decisions ordinarily integrate information across multiple views, both eyes, and longitudinal examinations. Although individual specialist labels and a collective label were provided, formal inter-rater reliability statistics and a fully reproducible consensus-adjudication procedure were not reported.

The absence of recommended patient-level development and test partitions also creates a potential risk of leakage if images from the same infant are allocated to different datasets. FARFUM-RoP should therefore be regarded as a useful exploratory development resource rather than a deployment-grade benchmark. Downstream studies should use patient-level separation, retain ungradable images, evaluate image-quality failure explicitly, and validate results in independent centres and populations.

##### Geographic and Device Generalisability

The included evidence originated from several geographic and technological contexts, including India, China, Iran, Mexico, Argentina, Hungary, Spain, the USA, Singapore, and Korea, and used imaging systems ranging from RetCam platforms to multiple smartphone configurations. However, this geographic diversity should not be interpreted as evidence of transportability. Independent cross-site, cross-device, and temporal validation was uncommon, and most studies were conducted within one programme, centre, country, or restricted population. Consequently, the respective effects of geography, device type, operator experience, sampling, and dataset size could not be separated reliably. Broader multicentre validation is required before assuming that performance will transfer across populations, capture systems, and service settings [53,56,58,60,61].

### 3.4. Methodological and Reporting Quality

The author-adapted six-domain quality rubric informed by APPRAISE-AI was applied to 11 studies that developed or evaluated AI models for clinical decision support. The public dataset descriptor and the non-randomised treatment-effect study were assessed separately using design-appropriate frameworks. Full study-level and domain-level results are presented in Supplementary Materials (S2).

Total scores ranged from 18 to 29 out of 30, with a median of 26 and a mean of 25.5. Clinical relevance was uniformly high, with all 11 studies scoring 5 out of 5. Reporting quality was also comparatively strong, with a mean of 4.64 and a median of 5. Methodological conduct and data quality had mean scores of 4.27 and 4.18, respectively. The robustness and generalisability domain was the lowest-scoring domain, with a mean of 3.64, followed by reproducibility and transparency, with a mean of 3.73.

The relatively high total scores require cautious interpretation. They were frequently driven by clearly defined clinical questions, clinically important target conditions, and generally complete reporting. They did not indicate that the studies were at low risk of bias or that the evaluated systems were externally valid, calibrated, transportable, or ready for routine implementation.

Important limitations remained common, including single-centre or geographically restricted samples, small numbers of clinically important positive cases, enriched or case–control populations, limited independent external or temporal validation, incomplete calibration, unclear handling of multiple images or both eyes from the same child, unavailable code or model artefacts, and absent formal subgroup fairness analyses. The domain-level findings were therefore interpreted together with the design-specific risk-of-bias assessments, which were given greater weight when determining the strength of the conclusions.

### 3.5. Risk of Bias Assessment

Risk of bias was assessed for the 12 empirical studies using design-specific tools: 10 diagnostic-accuracy studies were assessed using QUADAS-AI, one prediction-model study using PROBAST + AI, and one non-randomised treatment-effect study using ROBINS-I. The FARFUM-RoP dataset descriptor was evaluated separately using an author-developed dataset-quality, representativeness, and applicability framework. Overall judgments: Among diagnostic studies, three of the ten diagnostic-accuracy studies were judged at high risk of bias, while seven had some concerns. None were judged at low risk. The prediction model carried some concerns. The non-randomised intervention study was judged to be at serious risk of bias (ROBINS-I), driven by confounding and outcome-measurement issues. The dataset descriptor raised some concerns regarding quality, representativeness, and applicability, reflecting representativeness, labelling, and documentation gaps.

Diagnostic accuracy (QUADAS-AI).

The dominant threats clustered in the index-test and flow/timing domains. Multiple studies blurred the separation between training, tuning, and evaluation or did not state patient-level splits clearly, creating a risk of overfitting and optimistically biassed performance estimates and, in one case, incorporation bias where clinician-derived information re-entered the index model. Calibration of probabilistic outputs was absent in all diagnostic studies, and formal subgroup or fairness analyses were not reported; only occasional, limited subgroup summaries appeared (e.g., age or developmental delay), with no parity analyses. Reference standards were usually acceptable (expert grading with adjudication in several), but masking and inter-rater reliability were inconsistently documented; one study relied on a single grader. Patient selection frequently reflected enriched or convenience samples (clinic-based, excluded low-quality images, or case–control designs), curtailing spectrum breadth and inflating apparent accuracy. Applicability was constrained by single-centre settings, device-specific pipelines, and narrow demographics; external or temporal validation was rare. Collectively, these features explain the concentration of “some concerns” judgments and the three high-risk ratings.

Prediction model (PROBAST + AI):

The model targeted 5-year high-myopia risk and demonstrated structured development with internal and limited external validation; predictors and outcomes were well defined. Nonetheless, the analysis domain raised some concerns: no calibration was reported; uncertainty was likely underestimated by bootstrapping at the eye level (ignoring within-child clustering); threshold derivation was opaque; and formal fairness/subgroup reporting was absent. Overall RoB: some concerns.

Non-randomised treatment (ROBINS-I):

The atropine-effectiveness study used an ML model as a pseudo-control. Risk was serious due to unmeasured confounding, use of both eyes per child without appropriate clustering, imperfect alignment of outcome measurement over time (cycloplegic vs. manifest refraction), absence of adherence data, and unquantified propagation of prediction error from the ML counterfactual into treatment-effect estimates.

Dataset-quality and applicability assessment:

The public ROP image dataset raised some concerns regarding dataset quality and applicability. These concerns reflected its single-centre origin, removal of low-quality images, image-level rather than examination-level labels, absence of reported formal inter-rater reliability statistics, limited provenance and versioning information, and lack of recommended patient-level development and test partitions. Its public accessibility was considered a strength, but it was interpreted as an exploratory development resource rather than a definitive deployment-grade benchmark.

Across evidence streams, three deficits recur: (i) limited external/temporal validation; (ii) absent calibration and no formal fairness assessment; and (iii) sampling/spectrum biases that restrict transportability.

### 3.6. Influence of Risk of Bias on the Strength of the Findings

Risk-of-bias findings materially reduced confidence in several apparently strong performance estimates. None of the diagnostic studies provided uniformly low-risk evidence, and most estimates arose from single-centre or enriched cohorts. Clinically important positive groups were frequently small, while independent external or temporal validation, calibration, and formal subgroup fairness analyses were uncommon.

Evidence supporting sensitivity-first ROP screening was therefore considered promising but uncertain. High sensitivity was sometimes achieved through permissive operating thresholds or patient-level aggregation and was accompanied by reduced specificity. Such operating points may be appropriate when missed disease carries severe consequences, but their clinical value depends on disease prevalence, image gradability, referral capacity, confirmatory pathways, and prospective monitoring.

Evidence favouring human–AI collaboration was also considered preliminary. The relevant studies were few, heterogeneous, and predominantly single centre. They demonstrated comparative performance under specific study conditions but did not establish improvements in time to treatment, visual outcomes, safety, or service efficiency across independent health systems.

Accordingly, the synthesis supports further prospective evaluation of AI-assisted pathways rather than routine autonomous deployment.

## 4. Discussion

Previous reviews of ophthalmic AI have predominantly synthesised adult conditions, broad technical applications, individual paediatric diseases, or specialist-centre workflows rather than examining how AI may operate across first-contact paediatric eye-care pathways [64,65,66,67,68,69]. By contrast, our review is paediatric-first and primary care-centric. Across indications ROP, retinoblastoma (RB), amblyopia risk, refractive error/myopia, congenital cataract, and paediatric acuity, we find that sensitivity-prioritised AI, task shifting, and tele-referral may support access within supervised pathways, although safety and clinical effectiveness remain insufficiently established.

The number of studies identified in the present review should be interpreted in relation to its deliberately focused care-context eligibility criteria. Jafarizadeh et al. [70] identified 84 original studies across the broader field of artificial intelligence in retinopathy of prematurity, encompassing retinal-image analysis, vascular-feature quantification, algorithm development, disease detection and classification, diagnosis, and prognosis. That review was designed to examine the wider technical and clinical ROP-AI literature and was not restricted to primary, community, task-shifted, tele-ophthalmology, or referral-linked pathways.

By contrast, the present review examined multiple paediatric ophthalmic conditions but included only studies with direct or explicit relevance to primary or community care, school or outreach screening, task-shifted acquisition, tele-ophthalmology, triage, surveillance, or referral between frontline and specialist services. Consequently, tertiary-centre and engineering studies reporting technical model performance without a defined primary/community workflow or referral-linked use case were outside the eligibility criteria.

The smaller evidence base therefore reflects a narrower translational and health-service question rather than the overall volume of technical AI research in ROP. This distinction also highlights the gap between algorithm development and evidence supporting implementation in real-world paediatric care pathways.

Adult autonomous DR AI sets the regulatory bar; paediatrics needs a different operating model. Pivotal multicentre primary-care trials for autonomous diabetic-retinopathy systems (IDx-DR; EyeArt) showed pre-specified thresholds, analysable-image quality gates, independent reference standards, and deployment-grade accuracy, enabling safe autonomy without on-site specialists [71,72]. Our paediatric corpus, by contrast, spans motion-prone infants, variable cooperation, heterogeneous devices, and narrower safety margins; here, some included studies reported higher performance for supervised human–AI workflows than for selected unaided or automated comparators; however, this evidence was limited and did not establish improved patient outcomes.

Established tele-ROP pathways provide a relevant safety and referral precedent for paediatric AI-assisted screening. More mature paediatric AI systems build on this safety logic by automating quality control and first-pass grading while preserving a “refer-when-uncertain” rule [56,60].

AAPOS/AAP instrument-based preschool screening [73] emphasises sensitivity, requires confirmatory examinations after a failed screen, and promotes uniform validation targets. Our amblyopia/photoscreener findings align with systems that explicitly escalate inconclusive outputs and may support a more conservative screening pathway, although comparative safety outcomes were not directly evaluated [57].

WHO’s *Vision & Eye Screening Implementation Handbook* promotes integrated, people-centred eye care through primary/community platforms, trained non-specialists, and stepwise escalation [74]. Our review found that commodity smartphones, nurse and teacher operators, and edge–cloud tele-referral operationalise this blueprint, positioning AI as a force multiplier inside WHO’s model rather than a standalone diagnostic revolution [53,58,60].

Interpreting performance through a primary-care lens, headline AUCs must be read against three realities. First, paediatric vision programmes face asymmetric harm: missing treatment-requiring disease in ROP or active RB is far costlier than over-referral, so systems deliberately tuned to minimise false negatives and that escalate ungradable or “inconclusive” outputs to referral are inherently more transferable to frontline use than devices optimised for higher specificity [52,56,57,60]. Second, two included studies reported higher performance for combined human–AI workflows than for their selected unaided or automated comparators: structured human-in-the-loop frameworks for paediatric acuity and RB surveillance captured cooperation-dependent edge cases while preserving AI speed and consistency, surpassing both unaided clinicians and end-to-end models [52,63]. Third, performance limitations appeared to arise from interacting factors, including sample size and class balance, data partitioning, aggregation procedures, geography, device characteristics, operator experience, and unmodelled within-child dependence [53,56,58,60,61].

Myopia and congenital cataract. A fundus-based DL system predicted 5-year high-myopia risk at AUC ≈0.93 (image-only) and ≈0.97 (with baseline refraction), while an ocular-appearance classifier using commodity cameras prospectively outperformed ophthalmologists for binary myopia detection [54,62]. Where imaging is impractical, a non-imaging ML model using historical and perinatal factors identified children at risk of congenital cataract with interpretable predictors suitable for primary care [59].

### 4.1. Feasibility and Scale Are Engineered, Not Inferred

Programmes that moved image acquisition to commodity hardware and shifted acquisition tasks to trained non-specialist operators demonstrated substantial throughput and operational feasibility, although patient-safety outcomes were not directly established. By contrast, the edge–cloud prototype’s modest accuracy cautions that cloud connectivity and model export do not substitute for balanced data and hardened QC.

### 4.2. Safety Is an Operational Property, Not a Single AUC

Safety cannot be inferred from AUC, sensitivity, or specificity alone. It depends on the complete clinical pathway, including image-quality control, prespecified operating thresholds, management of ungradable or uncertain outputs, specialist confirmation, and access to timely referral.

Several included systems deliberately prioritised sensitivity over specificity. This may be clinically appropriate for treatment-requiring retinopathy of prematurity or active retinoblastoma, where false-negative results may lead to severe and irreversible harm. However, lower specificity increases false-positive referrals and may place additional pressure on specialist services. The practical safety of a sensitivity-first model therefore depends not only on missed-disease risk but also on disease prevalence, image gradability, referral capacity, and the availability of confirmatory assessment.

The amblyopia-risk comparison illustrates the importance of conservative uncertainty handling. The established PlusoptiX system generated more inconclusive results than AI Optic, but treating inconclusive examinations as referrals preserved a more cautious screening pathway and was accompanied by better discrimination and refractive accuracy [57]. This finding supports explicit escalation of uncertain or ungradable outputs rather than automatically treating them as negative results.

Some studies also reported saliency or attention outputs that corresponded to clinically relevant anatomical regions, including posterior-pole vasculature in ROP and tumour-associated regions in retinoblastoma [52,60]. These findings indicate anatomical plausibility but do not establish that the explanations improved clinician decisions, reduced errors, or enhanced patient safety. Overall, the evidence supports prioritising the evaluation and procurement of systems that make uncertainty, image-quality failure, and escalation requirements transparent. Nevertheless, confidence remains limited because most studies were single-centre, external validation was uncommon, and few directly evaluated safety outcomes, referral consequences, or the clinical effect of explainability. These findings are consistent with AAPOS guidance favouring conservative referral after failed or inconclusive paediatric vision screening [73].

### 4.3. Equity, Transportability, and Openness

Evidence spans India, China, Iran, Mexico/Argentina, Hungary, Spain, the USA, Singapore, and Korea, and multiple platforms from RetCam to diverse smartphones; QC-aware pipelines tended to preserve accuracy across settings. However, formal fairness analyses were essentially absent, and external or temporal validation was uncommon—both are key risks to equitable scale. The public ROP dataset [55] is a useful contribution to open ROP dataset availability, but its expert-curated, high-quality images also illustrate the tension between clean benchmarks and real-world variance. To avoid these problems, future paediatric AI studies should recruit beyond single-centre convenience samples; report subgroup performance (sex, disability, socioeconomic status, ethnicity); preserve “warts-and-all” images; and publish patient-level recommended splits to prevent leakage [55,60,61].

Evidence for economic value is limited. Across the included studies, only the retinoblastoma system reported a trial-embedded cost-utility analysis; for ROP, amblyopia/photoscreening, myopia screening, or prognostic models, no cost-effectiveness or budget-impact evaluations were found, so health-system value remains unknown.

The ingredients for positive value are present: orders-of-magnitude cheaper capture hardware (smartphones vs. wide-field cameras), population-scale throughput (hundreds screened per hour), and potential prevention of lifelong disability [52,58,60]. The missing are decision-grade economic evaluations (cost-utility, budget impact) embedded in pragmatic deployments that track downstream utilisation, time-to-treatment, and vision outcomes under different thresholds. None of the included studies reported performance stratified by sex, ethnicity, socioeconomic status, or disability. Without fairness analyses, it is impossible to know whether these algorithms disproportionately misclassify certain groups.

### 4.4. Strengths and Limitations

Strengths. This review is paediatric-first and primary/community care-centred, spanning ROP, retinoblastoma, amblyopia/myopia, congenital cataract, and paediatric acuity with a direct focus on frontline workflows (task-shifting, quality control, escalation). We used a comprehensive multi-database search across clinical and engineering sources and a transparent PRISMA-aligned process with dual independent screening/extraction, rater calibration, and full traceability to evidence. Methodologically, we combined PICo for index–context framing with SPIDER to capture operational outcomes, prioritised patient-level metrics, and avoided misleading pooling. Our AI-aware appraisal combined an adapted APPRAISE-AI instrument with design-specific risk-of-bias tools and explicitly considered clustering, aggregation-related inflation, and model opacity. We also assessed equity/transportability and *publicly available datasets*, recognising their role in scalable benchmarking.

Limitations. The evidence base is small and heterogeneous, precluding meta-analysis and limiting head-to-head comparability. Most studies are single-centre with narrow spectra, scarce external/temporal validation, inconsistent calibration, unclear unit-of-analysis choices, and lack subgroup and fairness reporting; these limitations risk inflating apparent accuracy and constrain generalisability. Although the adapted quality rubric indicated generally strong clinical framing and reporting, its summed scores should not be interpreted as measures of evidence certainty or implementation readiness, particularly because weaknesses in external validation, calibration, clustering, and representativeness may be concealed by stronger scores in other domains. Economic evaluations and patient-important outcomes (time-to-treatment, vision trajectories) were rarely reported, so health-system value remains uncertain. Although we searched multiple databases, unpublished or non-indexed evaluations may be missing. Several datasets and pipelines are device- or centre-specific, and some are expert-curated, potentially under-representing real-world image quality. Collectively, these constraints warrant cautious, evaluation-bound use and emphasise the need for multicentre, prospectively validated, calibrated, and equity-audited studies with embedded cost-effectiveness analyses. The deliberately narrow care-context criterion means that this review should not be interpreted as a comprehensive map of all technical AI research in paediatric ophthalmology or ROP. Laboratory-based and tertiary-centre studies without an explicit primary/community, screening, triage, tele-ophthalmology, surveillance, or referral-linked use case were outside its scope. The review was not prospectively registered, and no publicly accessible, time-stamped protocol was available; consequently, readers cannot independently verify that all methodological decisions were established before the review was conducted. This limitation is partly mitigated by the detailed reporting of the eligibility criteria, complete search strategies, screening and extraction procedures, and appraisal methods.

### 4.5. Implications for Policy and Practice

The evidence supports cautious, evaluation-bound exploration, not routine adoption of AI to expand paediatric eye care, given that most studies are single-centre, lack external or temporal validation, and rarely report calibration or subgroup/fairness performance. In neonatal units with retinopathy of prematurity (ROP) backlogs, a sufficiently validated smartphone or wide-field imaging system could be evaluated as a clinician-supervised triage layer within a controlled implementation programme, so ophthalmologists prioritise infants flagged for referrals. School and primary-care programmes can similarly integrate automated screening for refractive error and amblyopia risk during routine checks, with teachers or nurses operating devices after brief training. Early rollouts should adopt sensitivity-first settings treating ungradable or uncertain outputs as positive screens and retain human-in-the-loop verification until local performance is demonstrated. Clinical pathways must be explicit: failed AI screens prompt confirmatory examination and treatment; borderline or ungradable cases are escalated; and image-quality checks are enforced at the point of capture. Governance should mirror the conservatism that enabled the safe introduction of adult diabetic-retinopathy AI, with pre-market evidence thresholds (external/temporal validation, calibration, subgroup reporting) and post-market duties (drift monitoring, incident reporting, and rollback procedures).

### 4.6. Procurement and Governance Standards

Before any procurement beyond controlled evaluation pilots, purchasers should require independent external and temporal validation at the patient level, calibration with prespecified operating points, and stratified performance across devices, sites, and key demographic subgroups (fairness). Before procurement, programmes should require a context-specific economic evaluation (e.g., cost-utility and budget impact) because cost-effectiveness remains unproven, except in one single-centre retinoblastoma study. Models must be well-calibrated with pre-specified operating points and supported by decision-curve or net-benefit analyses tailored to local prevalence and service capacity, so thresholds align with operational constraints. A formal safety architecture is essential, mandating escalation of ungradable or uncertain cases, minimum quality-control yield targets (for example, acceptable gradability rates), and prospective false-negative audits during staged roll-out consistent with AAPOS-style conservatism. Fairness must be the default, with transparent subgroup performance (age bands, sex, ethnicity, device, site, comorbidity) and a concrete remediation plan where gaps are detected, including retraining, threshold adjustment, or targeted QC. Lifecycle governance should enforce model versioning and change logs, continuous post-market drift monitoring, and a clear pause/rollback mechanism when safety signals emerge. Contracts should hard-wire these expectations through subgroup performance guarantees, audit rights, and service-level agreements for updates and hot-fixes.

### 4.7. Economic Value

Falling capture-hardware costs, higher throughput, and the potential to avert lifelong visual disability suggest potential economic value, although cost-effectiveness remains unestablished. Early deployments should be embedded in pragmatic implementation studies that track downstream utilisation (referrals and confirmatory examinations), time-to-treatment, and vision outcomes, and that model budget impact across alternative operating thresholds. Given the steep social cost of missed paediatric blindness, value may accrue even with modest specificity; nonetheless, threshold selection should be co-designed with providers to avoid referral bottlenecks and diagnostic congestion.

### 4.8. Paediatric-Specific Evidence and Implementation Requirements

Adult diabetic-retinopathy systems provide important methodological and regulatory precedents, including prospective evaluation, standardised image-acquisition protocols, prespecified operating thresholds, image-quality controls, independent reference standards, and defined referral pathways [71,72]. However, evidence supporting these adult systems cannot be transferred directly to paediatric ophthalmology because paediatric applications span premature neonates, preverbal children, school-aged children, and adolescents, with substantial differences in anatomy, disease prevalence, developmental stage, cooperation, and imaging requirements [22,23].

Image acquisition is itself a major source of risk in paediatric care. Infant movement, variable fixation, crying, small pupils, limited cooperation, and operator-dependent capture can produce ungradable or incomplete examinations. Some conditions require serial assessment, multiple retinal views, integration across both eyes, or examination under anaesthesia. Performance observed using selected high-quality images may therefore overestimate reliability at the point of care, particularly when systems are transferred to non-specialist operators, different devices, or community and outreach settings [52,56,60].

The consequences of error are also condition-specific. Missing treatment-requiring ROP or active retinoblastoma may result in rapid and irreversible harm. Sensitivity-first thresholds may consequently be appropriate, but reduced specificity can increase false-positive referrals and place additional pressure on scarce specialist services. Paediatric implementation should therefore evaluate not only sensitivity and discrimination but also gradability, recapture rates, false-positive referral burden, time to confirmatory assessment, and the capacity of the receiving clinical pathway [52,56,60].

Paediatric AI systems consequently require validation across developmental stages, devices, operators, sites, and relevant clinical and demographic subgroups. Evaluation should include examination-level as well as image-level performance, explicit management of uncertain and ungradable outputs, caregiver communication, clearly defined responsibilities for human oversight, and post-deployment monitoring for acquisition failure, performance drift, and unequal subgroup effects. These requirements are particularly important because children may be underrepresented in datasets used for model development and may experience risks related to transferability, privacy, accountability, and long-term data use that differ from those encountered in adult populations [22,23,75].

Until these paediatric-specific requirements are satisfied, supervised human-in-the-loop implementation within explicit escalation and referral pathways is more defensible than unrestricted autonomous use.

### 4.9. Future Research Priorities

Future research should prioritise multicentre prospective evaluation of AI-enabled paediatric ophthalmic pathways. Studies should include independent external and, where feasible, temporal validation; prespecified operating thresholds; complete calibration assessment; and adequately powered subgroup analyses. Comparative designs may include cluster-randomised trials, stepped-wedge implementation studies, or pragmatic prospective evaluations, depending on the clinical setting and maturity of the technology. Outcomes should extend beyond diagnostic discrimination to include detection rates, false-negative events, referral completion, time to confirmatory assessment, time to treatment, visual outcomes, and adverse workflow consequences. Open paediatric datasets should include recommended patient-level development and test partitions, images acquired across multiple devices and sites, and representative examples of poor-quality and ungradable examinations. Transparent evaluation code, model-version information, and standardised reporting of image-quality failure would facilitate reproducible comparison and independent validation. Benchmark performance should be reported at clinically relevant frame-, image-, eye-, examination-, and patient-level units, with aggregation rules and handling of within-child correlation clearly specified.

Calibration and fairness should be treated as core evaluation components rather than optional supplements to discrimination metrics. Studies should report calibration across clinically relevant risk ranges, prespecify operating points, and evaluate performance across developmental stages, sex, relevant racial or ethnic groups, disability, comorbidity, device, operator, and site. Reporting should follow the applicable AI-specific guidance.

Explainability research should move beyond the visual presentation of generic saliency maps. Future studies should determine whether explanations are stable, clinically meaningful, and capable of improving reader decisions, error detection, escalation, or communication with patients and caregivers. Explanation methods should be evaluated prospectively rather than assumed to enhance safety on the basis of anatomical plausibility alone.

Finally, implementation and economic studies should evaluate the longitudinal effects of AI on clinic flow, staffing, training, recapture, referral volume, specialist workload, time to treatment, and avoidable visual impairment. Cost-effectiveness and budget-impact analyses should compare alternative operating thresholds and service-delivery models and should be reported using established health-economic guidance. These evaluations should provide decision-grade evidence for procurement, reimbursement, and coverage while accounting for local disease prevalence, workforce capacity, infrastructure, and referral constraints.

## 5. Conclusions

The available evidence indicates that AI may support selected paediatric ophthalmic screening, triage, diagnosis, and surveillance tasks when used within quality-controlled and human-supervised pathways. However, confidence in the reported performance is constrained by single-centre designs, small numbers of clinically important cases, enriched samples, heterogeneous analytical units, potentially optimistic aggregation rules, limited external or temporal validation, incomplete calibration, and absent fairness assessment.

Sensitivity-first thresholds may be appropriate when false negatives carry severe consequences, but they can generate substantial false-positive referral burdens and should not be interpreted as evidence of safety in isolation. Early findings supporting human–AI collaboration are encouraging but do not yet demonstrate improved patient outcomes across independent settings.

Routine autonomous implementation is therefore premature. Future studies should provide multicentre prospective validation, patient-level data separation and analysis, transparent aggregation rules, full calibration, subgroup evaluation, explicit management of ungradable examinations, and embedded clinical and economic outcomes.

## Figures and Tables

**Table 1:** PICo framing.

Element	Operational Definition for This Review
Population (P)	Children and adolescents aged 0–18 years for whom ophthalmic screening, triage, surveillance, diagnosis, prognostication, or treatment evaluation was relevant. Studies including adults older than 18 years were eligible only when paediatric data were separately extractable.
Interest (I)	AI-enabled tools (ML/DL/algorithmic decision support), imaging (e.g., fundus photographs/videos, ocular-appearance images, photoscreeners) or non-imaging models (e.g., risk prediction). This category included autonomous and human-in-the-loop workflows and on-device or edge–cloud implementations.
Context (Co)	(a) Direct primary/community care (schools, community clinics, NICU outreach, tele-ophthalmology “spokes”), or (b) primary care-relevant evaluations in specialist/research settings that directly enable primary/community workflows (e.g., screening, triage, surveillance hand-offs) or release public datasets explicitly intended to train/benchmark such tools.

**Table 2:** Sample, Phenomenon of Interest, Design, Evaluation, and Research type (SPIDER) components.

SPIDER Element	Operationalisation in This Review
Sample (S)	Children and adolescents aged 0–18 years; studies including adults older than 18 years were eligible only when data for participants aged ≤18 years were separately extractable.
Phenomenon of Interest (PI)	AI use within paediatric eye-care workflows that intersect primary/community care (screening, triage, surveillance, task-shifting, tele-referral, quality control, escalation logic).
Design (D)	Prospective/retrospective clinical evaluations; head-to-head device studies; development/validation with a prespecified test/validation set or a prospective cohort; public dataset descriptors explicitly enabling primary/community screening/surveillance.
Evaluation (E)	Diagnostic/prognostic metrics (Se/Sp at patient level, AUC, PPV/NPV, calibration), operational/safety outcomes (QC/gradability yield, inconclusive rate and escalation, throughput/time). Patient-level outcomes (e.g., detection of treatment-requiring disease, referral decision), and economic signals (costs, ICER/ICUR). Interpretability was recorded as a supportive outcome.
Research type (R)	Quantitative clinical evaluations (diagnostic accuracy, prediction, quasi-experimental treatment evaluation), plus operational feasibility/implementation reports that were peer-reviewed.

**Table 3:** Inclusion and exclusion criteria.

Domain	Inclusion Criteria	Exclusion Criteria
Population	Children and adolescents aged 0–18 years. Studies including participants older than 18 years were eligible only when data for those aged ≤18 years were separately extractable.	Adult-only populations; mixed-age studies in which data for participants aged ≤18 years were not separately extractable.
Clinical domain	Ophthalmology (screening, triage, diagnosis, prognostication, surveillance, or treatment evaluation).	Non-ophthalmic focus.
Intervention (AI)	AI-enabled tool (ML/DL/algorithmic decision support), imaging (fundus, ocular appearance, video, photoscreeners) or non-imaging (risk models); autonomous or human-in-the-loop; on-device or edge–cloud.	No AI component; purely heuristic rules without any trained algorithm.
Care context	(a) Direct primary/community care (schools, community clinics, NICU outreach, tele-ophthalmology “spokes”), or (b) primary care-relevant evaluations in secondary/tertiary or research settings that directly enable primary/community workflows (screening/triage/surveillance hand-offs, tele-referral/task-shifting) or provide public datasets explicitly built to train/benchmark such tools.	Hospital/tertiary studies without an explicit primary/community workflow, hand-off, or screening/surveillance intent; datasets with no stated primary-care use-case.
Comparator	Any: clinicians/usual care, validated devices, reader consensus, historical or AI counterfactual (e.g., natural-history model); none acceptable for data descriptors explicitly enabling primary/community screening or surveillance.	Comparators that are irrelevant to eye care; studies where comparator is undefined and not a data descriptor.
Outcomes	≥1 diagnostic/prognostic metric (sensitivity, specificity, AUC, calibration, PPV/NPV), patient-level outcome (e.g., treatment-requiring disease detected, referral decision), operational metric (throughput, QC yield, inconclusive rate), or economic outcome (costs, ICER/ICUR). Interpretability/explainability accepted as supportive.	No extractable outcomes (neither performance, operational, nor economic); narrative/opinion without data.
Study types	Prospective/retrospective clinical evaluations; head-to-head device studies; development/validation with a prespecified test/validation set or prospective cohort; data descriptors explicitly intended to support primary/community screening/surveillance.	Protocols without results; editorials/opinions; pure engineering/bench work without stated primary-care screening/surveillance relevance.

**Table 5:** Analytical units, aggregation procedures, clustering concerns, and implications for the reliability of reported performance.

Study	Model-Input or Observation Unit	Primary Performance-Reporting Unit	Aggregation or Decision Rule	Clustering or Dependence Concern	Likely Effect on Reliability and Interpretation
K. Zhang et al., 2020 [63]	Individual video frames and sequential visual-acuity examination responses	Examination-level end-to-end accuracy	Frame-level detections were combined with rule-based modules and clinician-informed decision logic to generate the final examination result	Multiple observations arose from the same examination, and the reported result reflected the complete multistage pipeline rather than independent frame classifications	The final accuracy estimate is clinically relevant but cannot be attributed solely to the deep-learning classifier; performance reflects frame processing, rule-based integration, and human-in-the-loop logic
Yang et al., 2020 [62]	Ocular-appearance images from individual eyes	Eye-level diagnostic performance	Three-angle ocular images were combined into a stitched image before classification	Both eyes could contribute observations, while patient-level separation between development and evaluation datasets was not reported clearly	If fellow eyes from the same child were split across datasets or analysed as independent observations, model performance and precision may have been overestimated
Csizek et al., 2023 [61]	Responses to multiple stereovision component tests	Child-level amblyopia or amblyogenic-risk classification	Outputs from several stereovision tests were combined using optimised perceptron-derived weights to produce a composite classification score	Repeated test measurements were nested within children, and not all component tests were completed by every participant	The high performance represents the complete composite testing procedure; incomplete component testing and optimisation of test weights may limit reproducibility outside the evaluated protocol
Young et al., 2023 [60]	Multiple smartphone fundus images from one or both eyes	Eye- and infant-level ROP classification	Image-level predictions were summarised across the available images, including median-based aggregation for higher-level classification	Multiple images and both eyes were nested within infants; the small number of treatment-requiring ROP cases further limited precision	Aggregation may stabilise predictions but can mask image-level heterogeneity; infant-level sensitivity should therefore be interpreted together with specificity, case numbers, and the underlying image-level results
Ortiz et al., 2025 [56]	Individual frames selected from smartphone fundus videos	Frame- and patient-level ROP performance	Selected frame predictions were combined to generate a patient-level screening decision, with a positive frame contributing to a positive patient classification	Multiple frames were nested within videos and patients, producing correlated observations	Patient-level aggregation increased sensitivity from 76.7% to 93.3% but reduced specificity to 55.2%; the patient-level estimate therefore reflects the complete frame-selection, aggregation, and thresholding strategy rather than improved discrimination of each frame
Foo et al., 2023 [54]	Multiple fundus images from individual eyes	Eye-level 5-year high-myopia prediction	Image features and clinical predictors were combined to generate an eye-level risk estimate	Both eyes from many children were included, while confidence intervals were generated using eye-level bootstrap resampling	Failure to resample or model at the child level may underestimate uncertainty because fellow-eye observations are correlated; discrimination estimates remain informative, but their reported precision may be optimistic
R. Zhang et al., 2023 [52]	Multiple retinal images from individual eyes	Eye-level retinoblastoma activity classification	The maximum image-level probability was used to assign the eye-level classification	Multiple images were nested within each eye and patient	Maximum-probability aggregation may improve sensitivity by allowing any strongly positive image to determine the eye-level result, but the estimate reflects the aggregation rule and may not represent typical individual-image performance
Han et al., 2025 [51]	Repeated refractive measurements from individual eyes	Eye-level treatment-effect estimate	Observed progression was compared with an ML-derived natural-history prediction for each eye	Both eyes from many children contributed observations, and within-child correlation was not adequately incorporated into the treatment-effect analysis	The effective sample size may have been overstated and confidence in the estimated treatment effect may be greater than warranted; prediction error from the ML counterfactual introduces additional uncertainty
Akbari et al., 2024, FARFUM-RoP [55]	Individual retinal images nested within infants and examinations	Image-level dataset labels; no model-performance estimate	Images were labelled individually as normal, pre-plus, or plus disease; no recommended patient-level development and test partition was provided	Multiple images from the same infant are correlated and could be allocated across development and test sets in downstream studies	Random image-level splitting could produce patient leakage and overly optimistic model performance; the dataset is more appropriate for exploratory development than for definitive independent benchmarking

Notes: The table includes studies for which the unit of analysis, aggregation procedure, or within-child dependence materially affected interpretation. “Reporting unit” refers to the level at which the principal performance estimate or clinical outcome was presented. Aggregated patient- or eye-level results are not inherently biassed when they correspond to the intended clinical decision; however, their validity depends on prespecified rules, patient-level data separation, appropriate handling of correlated observations, and simultaneous reporting of sensitivity, specificity, and uncertainty. FARFUM-RoP is included because its image-within-infant structure has direct implications for the validity of downstream model evaluation, although the report itself did not evaluate an AI model. Abbreviations: ML, machine learning; ROP, retinopathy of prematurity.

## Data Availability

The data generated and analysed during this study are included in this published article and its Supplementary Information. Additional materials are available from the corresponding author upon reasonable request.

## Supplementary Materials

The following supporting information can be downloaded at: https://www.mdpi.com/article/10.3390/diagnostics16152389/s1. Supplementary Materials: PRISMA 2020 Checklist. Table S1a. Databases, coverage, and core search construction; Table S1b. Database-Specific Search Strategies; Table S2a. Methodological and reporting quality assessment using an author-adapted six-domain rubric informed by APPRAISE-AI; Table S2b. Domain-specific scoring anchors for the author-adapted six-domain rubric; Table S2c: Summary of Domain and Total Scores; Table S3a. Author-adapted QUADAS-2 risk-of-bias assessment incorporating QUADAS-AI–informed AI-specific considerations; Table S3b. Summary PROBAST+AI assessment of the prediction-model study;Table S3c. Risk-of-Bias Assessment Using ROBINS-I: Non-Randomised Treatment-Effect Study; Table S3d. Dataset Quality, Representativeness, and Applicability Assessment of FARFUM-RoP.

## Abbreviations

Abbreviation	Full Term
AAP	American Academy of Pediatrics
AAPOS	American Association for Pediatric Ophthalmology and Strabismus
AI	Artificial intelligence
APPRAISE-AI	Quantitative evaluation tool for clinical AI decision-support studies
AUC	Area under the receiver operating characteristic curve
CI	Confidence interval
CNN	Convolutional neural network
CONSORT-AI	Consolidated Standards of Reporting Trials—AI extension
DLA-RB	Deep-learning assistant for retinoblastoma
DL	Deep learning
DR	Diabetic retinopathy
EUA	Examination under anaesthesia
EyeArt	Autonomous AI system for diabetic-retinopathy screening
FARFUM-RoP	Public retinopathy-of-prematurity posterior-pole image dataset
FDA	(US) Food and Drug Administration
GP	General practitioner
HIL	Human-in-the-loop
ICER	Incremental cost-effectiveness ratio
ICUR	Incremental cost-utility ratio
IDx-DR	Autonomous AI system for diabetic-retinopathy detection
ML	Machine learning
NICU	Neonatal intensive care unit
NPV	Negative predictive value
PICo	Population–Interest–Context (question-framing tool)
PPV	Positive predictive value
PRISMA	Preferred Reporting Items for Systematic Reviews and Meta-Analyses
PROBAST + AI	Prediction model Risk Of Bias ASsessment Tool—AI extension
QC	Quality control
QUADAS-AI	Quality Assessment of Diagnostic Accuracy Studies—AI extension
RB	Retinoblastoma
RCT	Randomised controlled trial
ResNet	Residual neural network architecture
ROC	Receiver operating characteristic
ROP	Retinopathy of prematurity
SBFI	Smartphone-based fundus imaging
Se	Sensitivity
Sp	Specificity
SPIDER	Sample, Phenomenon of Interest, Design, Evaluation, Research type (question-framing tool)
STARD-AI	Standards for Reporting Diagnostic Accuracy Studies—AI extension
TR-ROP	Treatment-requiring retinopathy of prematurity
TRIPOD-AI	Transparent Reporting of a multivariable prediction model for Individual Prognosis Or Diagnosis—AI extension
WHO	World Health Organization
ABCD ellipsoid	Vector metric summarising refractive error accuracy in photoscreener comparisons
