# Supplementary material for: Clinical Performance and Implementation of AI-Enabled Paediatric Ophthalmic Screening, Triage, Diagnosis, and Surveillance in Primary, Community, and Referral-Linked Pathways: A Systematic Review
Source: Diagnostics (Basel). 2026 Jul 29;16(15):2389. doi: 10.3390/diagnostics16152389 (PMC13465666; doi:10.3390/diagnostics16152389)
Supplement: Supplementary file 1 [file diagnostics-16-02389-s001.zip › PRISMA_2020_checklist RA.pdf]

Section and Topic	Item #	Checklist item	Location where item is reported
**TITLE**			
Title	1	Identify the report as a systematic review.	Title, manuscript p. 1.
**ABSTRACT**			
Abstract	2	See the PRISMA 2020 for Abstracts checklist.	Structured Abstract, manuscript pp. 1–2.
**INTRODUCTION**			
Rationale	3	Describe the rationale for the review in the context of existing knowledge.	Introduction, manuscript pp. 2–4, particularly the evidence-gap and implementation-rationale paragraphs.
Objectives	4	Provide an explicit statement of the objective(s) or question(s) the review addresses.	Sections 1.1 (Aim) and 1.2 (Objectives), manuscript p. 4.
**METHODS**			
Eligibility criteria	5	Specify the inclusion and exclusion criteria for the review and how studies were grouped for the syntheses.	Sections 2.1–2.2 and Tables 1–3, manuscript pp. 4–7; study grouping for thematic synthesis is described in Section 2.5, manuscript pp. 7–8.
Information sources	6	Specify all databases, registers, websites, organisations, reference lists and other sources searched or consulted to identify studies. Specify the date when each source was last searched or consulted.	Section 2.3, manuscript p. 7; Appendix 1A, pp. 1–2; Appendix 1B, pp. 3–5. Five databases/platforms and the final search date (30 March 2026) are reported. Section 3.1, manuscript pp. 9–10, reports that no additional records were identified through other sources.
Search strategy	7	Present the full search strategies for all databases, registers and websites, including any filters and limits used.	Appendix 1B, pp. 3–5. Complete database-specific strategies are provided for MEDLINE/PubMed, Embase/Ovid, Web of Science Core Collection, Scopus, and IEEE Xplore, including search dates, field syntax, limits, and records retrieved.
Selection process	8	Specify the methods used to decide whether a study met the inclusion criteria of the review, including how many reviewers screened each record and each report retrieved, whether they worked independently, and if applicable, details of automation tools used in the process.	Abstract, manuscript p. 1, and Section 2.4, manuscript p. 7. Rayyan was used to manage screening; two reviewers worked independently, with disagreements resolved by discussion or a third reviewer. No automated eligibility-decision tool was reported.
Data collection process	9	Specify the methods used to collect data from reports, including how many reviewers collected data from each report, whether they worked independently, any processes for obtaining or confirming data from study investigators, and if applicable, details of automation tools used in the process.	Section 2.5, manuscript p. 7, reports independent extraction by two reviewers using a predefined framework and third-reviewer adjudication.
Data items	10a	List and define all outcomes for which data were sought. Specify whether all results that were compatible with each outcome domain in each study were sought (e.g.,, for all measures, time points, analyses), and if not, the methods used to decide which results to collect.	Tables 1 and 3, manuscript pp. 4–6, and Section 2.5, manuscript pp. 7–8. Diagnostic/prognostic, operational/safety, patient-level, interpretability, and economic outcomes are specified; where several thresholds were available, the operational or prespecified sensitivity-first threshold was prioritised.
	10b	List and define all other variables for which data were sought (e.g., participant and intervention characteristics, funding sources). Describe any assumptions made about any missing or unclear information.	Section 2.5, manuscript pp. 7–8, lists study setting, care context, condition, population/sample, model, inputs, comparator, deployment, workflow, limitations, analytical units, data partitioning, aggregation, both-eye inclusion, and clustering.
Study risk of bias assessment	11	Specify the methods used to assess risk of bias in the included studies, including details of the tool(s) used, how many reviewers assessed each study and whether they worked independently, and if applicable, details of automation tools used in the process.	Section 2.7, manuscript pp. 8–9; Appendix 3A, pp. 1–3; Appendix 3B, p. 4; Appendix 3C, p. 5; Appendix 3D, pp. 6–7. Design-specific tools/frameworks, reviewer independence, consensus, and third-reviewer adjudication are reported
Effect measures	12	Specify for each outcome the effect measure(s) (e.g., risk ratio, mean difference) used in the synthesis or presentation of results.	Tables 1 and 3, manuscript pp. 4–6, and Section 2.5, manuscript pp. 7–8. Measures presented include sensitivity, specificity, AUC, PPV/NPV, calibration, throughput/quality-control measures, referral outcomes, and economic measures. No pooled effect measure was used.
Synthesis methods	13a	Describe the processes used to decide which studies were eligible for each synthesis (e.g., tabulating the study intervention characteristics and comparing against the planned groups for each synthesis (item #5)).	Sections 2.1–2.2, manuscript pp. 4–7, and Section 2.5, manuscript pp. 7–8. PICo/SPIDER framing, eligibility criteria, care context, clinical task, and thematic domains determined which studies contributed to each synthesis.
	13b	Describe any methods required to prepare the data for presentation or synthesis, such as handling of missing summary statistics, or data conversions.	Section 2.5, manuscript pp. 7–8, and Section 3.3.1.4/Table 5, manuscript pp. 21–24. The review describes threshold prioritisation and the handling/interpretation of frame-, image-, eye-, examination-, and patient-level results, aggregation, and within-child clustering. No imputation or numerical data conversion was reported as necessary.
	13c	Describe any methods used to tabulate or visually display results of individual studies and syntheses.	Section 2.5, manuscript pp. 7–8. Study characteristics and findings are displayed in Table 4, manuscript pp. 12–19; analytical-unit/aggregation issues in Table 5, manuscript pp. 22–24; methodological quality in Appendix 2, pp. 1–5; and risk of bias in Appendix 3, pp. 1–7.
	13d	Describe any methods used to synthesise results and provide a rationale for the choice(s). If meta-analysis was performed, describe the model(s), method(s) to identify the presence and extent of statistical heterogeneity, and software package(s) used.	Section 2.5, manuscript pp. 7–8. A thematic synthesis structured by PICo and SPIDER was used. Statistical pooling was not attempted because of heterogeneity in thresholds, analytical units, settings, operators, and hardware.

Section and Topic	Item #	Checklist item	Location where item is reported
	13e	Describe any methods used to explore possible causes of heterogeneity among study results (e.g., subgroup analysis, meta-regression).	Section 2.5, manuscript pp. 7–8. Potential sources of heterogeneity were explored narratively through analytical unit, aggregation, clustering, setting, operator, device/hardware, geography, and validation context; no subgroup meta-analysis or meta-regression was conducted.
	13f	Describe any sensitivity analyses conducted to assess robustness of the synthesised results.	**NOT APPLICABLE/NOT CONDUCTED:** No sensitivity analysis was conducted because no meta-analysis or other statistical synthesis was performed.
Reporting bias assessment	14	Describe any methods used to assess risk of bias due to missing results in a synthesis (arising from reporting biases).	**NOT REPORTED:** No method was described for assessing risk of bias due to missing results in a synthesis (reporting or publication bias).
Certainty assessment	15	Describe any methods used to assess certainty (or confidence) in the body of evidence for an outcome.	**NOT FORMALLY PERFORMED:** No certainty-of-evidence framework (for example, GRADE) was used. Section 2.6, manuscript p. 8, explicitly states that the quality-rubric scores were not interpreted as measures of evidence certainty.
**RESULTS**			
Study selection	16a	Describe the results of the search and selection process, from the number of records identified in the search to the number of studies included in the review, ideally using a flow diagram.	Section 3.1 and Figure 1, manuscript pp. 9–10; Appendix 1B, p. 5. Database-specific counts, duplicate removal, screening, full-text assessment, exclusions, and 13 included records are reported.
	16b	Cite studies that might appear to meet the inclusion criteria, but which were excluded, and explain why they were excluded.	**NOT REPORTED:** Section 3.1 and Figure 1, manuscript pp. 9–10, provide aggregate full-text exclusion categories and counts, but do not cite individual apparently eligible reports with study-specific reasons for exclusion.
Study characteristics	17	Cite each included study and present its characteristics.	Section 3.2, manuscript p. 11, and Table 4, manuscript pp. 12–19. Each of the 13 included records is cited and its setting, population, target condition, model/input, performance, deployment context, findings, and limitations are presented.
Risk of bias in studies	18	Present assessments of risk of bias for each included study.	Section 3.5, manuscript pp. 27–28; Appendix 3A, pp. 1–3; Appendix 3B, p. 4; Appendix 3C, p. 5; Appendix 3D, pp. 6–7. Study-level/domain-level judgments and key concerns are presented for all evidence streams.
Results of individual studies	19	For all outcomes, present, for each study: (a) summary statistics for each group (where appropriate) and (b) an effect estimate and its precision (e.g., confidence/credible interval), ideally using structured tables or plots.	Table 4, manuscript pp. 12–19, and Sections 3.3.1–3.3.4, manuscript pp. 20–27, present study-level samples and principal performance/effect estimates. Measures of precision are provided only where reported or selected for discussion
Results of syntheses	20a	For each synthesis, briefly summarise the characteristics and risk of bias among contributing studies.	Sections 3.3–3.6, manuscript pp. 20–29; Appendix 2, pp. 1–5; Appendix 3, pp. 1–7. Characteristics, methodological/reporting quality, risk of bias, and their influence on confidence are summarised for contributing studies.
	20b	Present results of all statistical syntheses conducted. If meta-analysis was done, present for each the summary estimate and its precision (e.g. confidence/credible interval) and measures of statistical heterogeneity. If comparing groups, describe the direction of the effect.	**NOT APPLICABLE:** No meta-analysis or other statistical synthesis was conducted. Results of the narrative thematic syntheses are presented in Section 3.3, manuscript pp. 20–27.
	20c	Present results of all investigations of possible causes of heterogeneity among study results.	Section 3.3.1.4 and Table 5, manuscript pp. 21–24; Section 3.3.4.2, manuscript p. 27; Sections 3.4–3.6, manuscript pp. 27–29. Variation related to analytical unit, aggregation, clustering, device, setting, geography, validation, and risk of bias is presented narratively.
	20d	Present results of all sensitivity analyses conducted to assess the robustness of the synthesised results.	**NOT APPLICABLE/NOT CONDUCTED:** No sensitivity analyses were conducted.
Reporting biases	21	Present assessments of risk of bias due to missing results (arising from reporting biases) for each synthesis assessed.	No formal assessment of bias due to missing results, selective non-reporting, or publication bias is presented for any synthesis.
Certainty of evidence	22	Present assessments of certainty (or confidence) in the body of evidence for each outcome assessed.	No certainty-of-evidence framework was applied. Section 3.6, manuscript pp. 28–29, provides a narrative explanation of how risk of bias reduced confidence in the findings.
**DISCUSSION**			
Discussion	23a	Provide a general interpretation of the results in the context of other evidence.	Discussion, manuscript pp. 29–35, particularly Sections 4.1–4.3; Conclusions, manuscript p. 35.
	23b	Discuss any limitations of the evidence included in the review.	Section 3.6, manuscript pp. 28–29, and Section 4.4, manuscript pp. 32–33.
	23c	Discuss any limitations of the review processes used.	Section 4.4, manuscript pp. 32–33, including limitations related to scope, heterogeneity, unpublished/non-indexed evidence, non-registration, and the absence of a publicly accessible time-stamped protocol.

Section and Topic	Item #	Checklist item	Location where item is reported
	23d	Discuss implications of the results for practice, policy, and future research.	Sections 4.5–4.9, manuscript pp. 33–35, covering practice/policy, procurement/governance, economic value, paediatric-specific implementation requirements, and future research.
**OTHER INFORMATION**			
Registration and protocol	24a	Provide registration information for the review, including register name and registration number, or state that the review was not registered.	Section 2.1, manuscript p. 4, and Section 4.4, manuscript p. 32: the review was not prospectively registered in PROSPERO or another systematic-review registry.
	24b	Indicate where the review protocol can be accessed, or state that a protocol was not prepared.	Section 4.4, manuscript p. 32, states that no publicly accessible, time-stamped protocol was available.
	24c	Describe and explain any amendments to information provided at registration or in the protocol.	The review was not registered and no publicly accessible protocol was available, so no registration/protocol amendments are described.
Support	25	Describe sources of financial or non-financial support for the review, and the role of the funders or sponsors in the review.	Funding statement, manuscript p. 36: no specific grant was received from any public, commercial, or not-for-profit funding agency; consequently, no funder role applied.
Competing interests	26	Declare any competing interests of review authors.	Conflicts of Interest statement, manuscript p. 36: the authors declare that they have no competing interests.
Availability of data, code and other materials	27	Report which of the following are publicly available and where they can be found: template data collection forms; data extracted from included studies; data used for all analyses; analytic code; any other materials used in the review.	Data Availability Statement, manuscript p. 36; Appendix 1, pp. 1–5; Appendix 2, pp. 1–8; Appendix 3, pp. 1–7. Search strategies, quality-assessment materials, and risk-of-bias assessments are supplied.

From: Page MJ, McKenzie JE, Bossuyt PM, Boutron I, Hoffmann TC, Mulrow CD, et al. The PRISMA 2020 statement: an updated guideline for reporting systematic reviews. BMJ 2021;372:n71. doi: 10.1136/bmj.n71. This work is licenced under CC BY 4.0. To view a copy of this licence, visit https://creativecommons.org/licenses/by/4.0/
