# Supplementary material for: Clinical Performance and Implementation of AI-Enabled Paediatric Ophthalmic Screening, Triage, Diagnosis, and Surveillance in Primary, Community, and Referral-Linked Pathways: A Systematic Review
Source: Diagnostics (Basel). 2026 Jul 29;16(15):2389. doi: 10.3390/diagnostics16152389 (PMC13465666; doi:10.3390/diagnostics16152389)
Supplement: Supplementary file 1 [file diagnostics-16-02389-s001.zip › S1.pdf]

S1

Table S1a. Databases, coverage, and core search construction.

Database & platform	Coverage window	Field tags & subject headings used	Core concept blocks combined	Filters/limits applied	Notes (examples of key terms per block)
MEDLINE (PubMed)	Inception–2026-03-30	MeSH + tiab	Paediatrics ∧ Ophthalmology ∧ AI/ML ∧ Primary/community	English; Humans;	MeSH: Infant, Child, Adolescent; Eye Diseases, Retinal Diseases, Retinoblastoma, Retinopathy of Prematurity, Amblyopia, Myopia; AI: Artificial Intelligence, Machine Learning, Deep Learning; textwords: "retina*", "ROP", "amblyop*", "myop*", "photoscreen*", "tele-oph*", "human-in-the-loop", "smartphone*".
Embase	Inception–2026-03-30	Emtree + ti,ab,kw	Paediatrics ∧ Ophthalmology ∧ AI/ML ∧ Primary/community	English; Humans; Article	Emtree: infant/, child/, adolescent/; eye disease/, retina/, retinoblastoma/, retinopathy of prematurity/, amblyopia/, myopia/; AI: artificial intelligence/, machine learning/, deep learning/, neural network/; context: primary health care/, telemedicine/, mass screening/.
Web of Science	1900–2026-03-30	TS (Topic)	Paediatrics ∧ Ophthalmology ∧ AI/ML ∧ Primary/community	English; Document types: Article/Review	TS examples: (infant* OR pediatric* OR paediatric*) AND (retina* OR "retinopathy of prematurity" OR amblyop* OR myop*) AND ("artificial intelligen*" OR "machine learning" OR "deep learning") AND (screen* OR triage OR "primary care" OR telemedicine OR photoscreen*).
Scopus	Inception–2026-03-30	TITLE-ABS-KEY	Paediatrics ∧ Ophthalmology ∧ AI/ML ∧ Primary/community	English; Doc types: Article/Review	TITLE-ABS-KEY mirrors WoS terms; includes deployment language: "edge computing", "mHealth", "task

					shifting", "quality control", "teleophthalmology".
IEEE Xplore (Journals only)	Inception–2026-03-30	"All Metadata" fields	Paediatrics ∧ Ophthalmology ∧ AI/ML ∧ Primary/community	English; Content type: Journals	Emphasises engineering phrasing: "computer vision", "convolutional", "transformer", "edge computing", "mobile app", combined with ophthalmic terms ("retinoblastoma", "ROP", "amblyopia") and pathway terms ("screening", "telehealth", "primary care").

Abbreviations: tiab = title/abstract; ti,ab,kw = title/abstract/keywords; TS = topic (title/abstract/keywords/Keywords Plus).

Table S1b. Database-Specific Search Strategies

Database, platform, and search date	Complete database-specific search strategy	Limits applied	Records retrieved*
**MEDLINE via PubMed \nSearched: 30 March 2026**	(("Infant, Newborn"[Mesh] OR "Infant"[Mesh] OR "Child"[Mesh] OR "Adolescent"[Mesh] OR newborn*[tiab] OR neonat*[tiab] OR infant*[tiab] OR preterm[tiab] OR prematur*[tiab] OR child*[tiab] OR pediatric*[tiab] OR paediatric*[tiab] OR schoolchild*[tiab] OR adolescen*[tiab]) AND ("Eye Diseases"[Mesh] OR "Vision Disorders"[Mesh] OR "Retinal Diseases"[Mesh] OR "Retinopathy of Prematurity"[Mesh] OR "Retinoblastoma"[Mesh] OR "Amblyopia"[Mesh] OR "Myopia"[Mesh] OR "Strabismus"[Mesh] OR "Refractive Errors"[Mesh] OR "Cataract"[Mesh] OR "Visual Acuity"[Mesh] OR ophthalm*[tiab] OR ocular[tiab] OR eye[tiab] OR vision[tiab] OR retina*[tiab] OR fundus[tiab] OR "retinopathy of prematurity"[tiab] OR ROP[tiab] OR retinoblastoma*[tiab] OR amblyop*[tiab] OR myop*[tiab] OR strabism*[tiab] OR "refractive error*"[tiab] OR "congenital cataract*"[tiab] OR "visual acuity"[tiab] OR photoscreen*[tiab] OR "plus disease"[tiab]) AND ("Artificial Intelligence"[Mesh] OR "Machine Learning"[Mesh] OR "Deep Learning"[Mesh] OR "Neural Networks, Computer"[Mesh] OR "Diagnosis, Computer-Assisted"[Mesh] OR "Image Processing, Computer-Assisted"[Mesh] OR "Decision Support Systems, Clinical"[Mesh] OR "artificial intelligence"[tiab] OR "machine learning"[tiab] OR "deep learning"[tiab] OR "neural network*"[tiab] OR "convolutional neural network*"[tiab] OR CNN[tiab] OR ResNet[tiab] OR "computer vision"[tiab] OR "computer-assisted diagnosis"[tiab] OR "automated detection"[tiab] OR "automated classification"[tiab] OR "automated screening"[tiab] OR "prediction model*"[tiab] OR "risk prediction"[tiab] OR "clinical decision support"[tiab] OR "human-in-the-loop"[tiab]) AND ("Primary Health Care"[Mesh] OR "Community Health Services"[Mesh] OR "Mass Screening"[Mesh] OR "School Health Services"[Mesh] OR "Telemedicine"[Mesh] OR "Referral and Consultation"[Mesh] OR "Mobile Applications"[Mesh] OR "Smartphone"[Mesh] OR screen*[tiab] OR diagnos*[tiab] OR detect*[tiab] OR classif*[tiab] OR triage[tiab] OR predict*[tiab] OR prognos*[tiab] OR surveill*[tiab] OR monitor*[tiab] OR "treatment effect*"[tiab] OR counterfactual[tiab] OR referral*[tiab] OR "primary care"[tiab] OR community[tiab] OR school*[tiab] OR telemedicine[tiab] OR telehealth[tiab] OR teleophthalmolog*[tiab] OR outreach[tiab] OR "task shifting"[tiab] OR nonspecialist*[tiab] OR nurse-led[tiab] OR technician-led[tiab] OR teacher-led[tiab] OR mHealth[tiab] OR smartphone*[tiab] OR "point of care"[tiab] OR remote[tiab] OR rural[tiab] OR NICU[tiab] OR workflow*[tiab] OR deploy*[tiab] OR implement*[tiab] OR feasib*[tiab] OR "image quality"[tiab] OR "quality control"[tiab] OR ungradable[tiab] OR inconclusive[tiab] OR dataset*[tiab] OR benchmark*[tiab]))	English; Humans; publication date to 30 March 2026	**39**
**Embase via Ovid \nSearched: 30 March 2026**	1. exp newborn/ OR exp infant/ OR exp child/ OR exp adolescent/ OR (newborn* OR neonat* OR infant* OR preterm OR prematur* OR child* OR pediatric* OR paediatric* OR schoolchild* OR adolescen*).ti,ab,kw. \n2. exp eye disease/ OR exp vision disorder/ OR exp retina disease/ OR exp retinopathy of prematurity/ OR exp retinoblastoma/ OR exp amblyopia/ OR exp myopia/ OR exp strabismus/ OR exp refractive error/ OR exp cataract/ OR exp visual acuity/ OR (ophthalm* OR ocular OR eye OR vision OR retina* OR fundus OR (retinopathy adj2 prematurity) OR ROP OR retinoblastoma* OR amblyop* OR myop* OR strabism* OR (refractive adj2 error*) OR (congenital adj2 cataract*) OR (visual adj2 acuity) OR photoscreen* OR (plus adj2 disease)).ti,ab,kw. \n3. exp artificial intelligence/ OR exp machine learning/ OR exp deep learning/ OR exp neural network/ OR exp computer assisted diagnosis/ OR exp image processing/ OR exp clinical decision support system/ OR ("artificial intelligence" OR "machine learning" OR "deep learning" OR neural network* OR convolutional neural network* OR CNN OR ResNet OR "computer vision" OR computer-assisted diagnos* OR automated detect* OR automated classif* OR automated screen* OR prediction model* OR risk prediction OR clinical decision support OR human-in-the-loop).ti,ab,kw. \n4. exp primary health care/ OR exp community care/ OR exp mass screening/ OR exp school health service/ OR exp telemedicine/ OR exp patient referral/ OR exp mobile application/ OR exp smartphone/ OR (screen* OR diagnos* OR detect* OR classif* OR triage OR predict* OR prognos* OR surveill* OR monitor* OR (treatment adj2 effect*) OR counterfactual OR referral*	English; Humans; Article; publication date to 30 March 2026	**46**

	OR primary care OR community OR school* OR telemedicine OR telehealth OR teleophthalmolog* OR outreach OR task shifting OR nonspecialist* OR nurse-led OR technician-led OR teacher-led OR mHealth OR smartphone* OR point of care OR remote OR rural OR NICU OR workflow* OR deploy* OR implement* OR feasib* OR image quality OR quality control OR ungradable OR inconclusive OR dataset* OR benchmark*).ti,ab,kw. \n5. 1 AND 2 AND 3 AND 4		
**Web of Science Core Collection, Clarivate \nSearched: 30 March 2026**	TS=(infant* OR newborn* OR neonat* OR preterm OR prematur* OR child* OR pediatric* OR paediatric* OR schoolchild* OR adolescen*) AND TS=(ophthalm* OR ocular OR eye OR vision OR retina* OR fundus OR (retinopathy NEAR/2 prematurity) OR ROP OR retinoblastoma* OR amblyop* OR myop* OR strabism* OR (refractive NEAR/2 error*) OR (congenital NEAR/2 cataract*) OR (visual NEAR/2 acuity) OR photoscreen* OR (plus NEAR/2 disease)) AND TS=("artificial intelligence" OR "machine learning" OR "deep learning" OR "neural network*" OR "convolutional neural network*" OR CNN OR ResNet OR "computer vision" OR "computer-assisted diagnosis" OR "automated detection" OR "automated classification" OR "automated screening" OR "prediction model*" OR "risk prediction" OR "clinical decision support" OR "human-in-the-loop") AND TS=(screen* OR diagnos* OR detect* OR classif* OR triage OR predict* OR prognos* OR surveill* OR monitor* OR (treatment NEAR/2 effect*) OR counterfactual OR referral* OR "primary care" OR community OR school* OR telemedicine OR telehealth OR teleophthalmolog* OR outreach OR "task shifting" OR nonspecialist* OR nurse-led OR technician-led OR teacher-led OR mHealth OR smartphone* OR "point of care" OR remote OR rural OR NICU OR workflow* OR deploy* OR implement* OR feasib* OR "image quality" OR "quality control" OR ungradable OR inconclusive OR dataset* OR benchmark*)	English; Article or Review; 1900–30 March 2026	**37**
**Scopus, Elsevier \nSearched: 30 March 2026**	TITLE-ABS-KEY(infant* OR newborn* OR neonat* OR preterm OR prematur* OR child* OR pediatric* OR paediatric* OR schoolchild* OR adolescen*) AND TITLE-ABS-KEY(ophthalm* OR ocular OR eye OR vision OR retina* OR fundus OR "retinopathy of prematurity" OR ROP OR retinoblastoma* OR amblyop* OR myop* OR strabism* OR "refractive error*" OR "congenital cataract*" OR "visual acuity" OR photoscreen* OR "plus disease") AND TITLE-ABS-KEY("artificial intelligence" OR "machine learning" OR "deep learning" OR "neural network*" OR "convolutional neural network*" OR CNN OR ResNet OR "computer vision" OR "computer-assisted diagnosis" OR "automated detection" OR "automated classification" OR "automated screening" OR "prediction model*" OR "risk prediction" OR "clinical decision support" OR "human-in-the-loop") AND TITLE-ABS-KEY(screen* OR diagnos* OR detect* OR classif* OR triage OR predict* OR prognos* OR surveill* OR monitor* OR "treatment effect*" OR counterfactual OR referral* OR "primary care" OR community OR school* OR telemedicine OR telehealth OR teleophthalmolog* OR outreach OR "task shifting" OR nonspecialist* OR nurse-led OR technician-led OR teacher-led OR mHealth OR smartphone* OR "point of care" OR remote OR rural OR NICU OR workflow* OR deploy* OR implement* OR feasib* OR "image quality" OR "quality control" OR ungradable OR inconclusive OR dataset* OR benchmark*) AND PUBYEAR < 2027	English; Article or Review	**52**
**IEEE Xplore Digital Library \nSearched: 30 March 2026**	(("All Metadata":infant* OR "All Metadata":newborn* OR "All Metadata":neonat* OR "All Metadata":preterm OR "All Metadata":prematur* OR "All Metadata":child* OR "All Metadata":pediatric* OR "All Metadata":paediatric* OR "All Metadata":adolescen*) AND ("All Metadata":ophthalm* OR "All Metadata":ocular OR "All Metadata":eye OR "All Metadata":vision OR "All Metadata":retina* OR "All Metadata":fundus OR "All Metadata":"retinopathy of prematurity" OR "All Metadata":ROP OR "All Metadata":retinoblastoma* OR "All Metadata":amblyop* OR "All Metadata":myop* OR "All Metadata":strabism* OR "All Metadata":"refractive error" OR "All Metadata":"congenital cataract" OR "All Metadata":"visual acuity" OR "All Metadata":photoscreen*) AND ("All Metadata":"artificial intelligence" OR "All Metadata":"machine learning" OR "All Metadata":"deep learning" OR "All Metadata":"neural network" OR "All Metadata":"convolutional neural network" OR "All Metadata":CNN OR "All Metadata":ResNet OR "All Metadata":"computer vision" OR "All Metadata":"computer-assisted diagnosis" OR "All Metadata":"automated detection" OR "All Metadata":"automated classification" OR "All Metadata":"automated screening" OR "All Metadata":"prediction model" OR "All Metadata":"clinical decision support" OR	English; Journals; publication year to 2026	**23**

	"All Metadata":"human-in-the-loop") AND ("All Metadata":screen* OR "All Metadata":diagnos* OR "All Metadata":detect* OR "All Metadata":classif* OR "All Metadata":triage OR "All Metadata":predict* OR "All Metadata":surveill* OR "All Metadata":monitor* OR "All Metadata":referral* OR "All Metadata":"primary care" OR "All Metadata":community OR "All Metadata":school* OR "All Metadata":telemedicine OR "All Metadata":telehealth OR "All Metadata":teleophthalmolog* OR "All Metadata":"task shifting" OR "All Metadata":smartphone* OR "All Metadata":"mobile health" OR "All Metadata":"edge computing" OR "All Metadata":"edge-cloud" OR "All Metadata":workflow* OR "All Metadata":implement* OR "All Metadata":"quality control" OR "All Metadata":dataset* OR "All Metadata":benchmark*))		
**Total identified before duplicate removal**	—	—	**197**
**Duplicate records removed**	—	—	**69**
**Unique records screened**	—	—	**128**

Searches were conducted from database inception to 30 March 2026. Controlled vocabulary was combined with database-specific free-text terms covering four concepts: paediatric populations, ophthalmic conditions or visual functions, artificial intelligence, and screening, diagnosis, triage, surveillance, prediction, treatment evaluation, or primary/community/referral-linked care. Terms within each concept were combined with OR, and the four concepts were combined with AND.

Abbreviations: AI, artificial intelligence; CNN, convolutional neural network; MeSH, Medical Subject Headings; NICU, neonatal intensive care unit; ROP, retinopathy of prematurity; TS, Topic field; tiab, title and abstract; ti,ab,kw, title, abstract, and keywords.
