# Supplementary material for: Clinical Performance and Implementation of AI-Enabled Paediatric Ophthalmic Screening, Triage, Diagnosis, and Surveillance in Primary, Community, and Referral-Linked Pathways: A Systematic Review
Source: Diagnostics (Basel). 2026 Jul 29;16(15):2389. doi: 10.3390/diagnostics16152389 (PMC13465666; doi:10.3390/diagnostics16152389)
Supplement: Supplementary file 1 [file diagnostics-16-02389-s001.zip › S2.pdf]

Table S2a. Methodological and reporting quality assessment using an author-adapted six-domain rubric informed by APPRAISE-AI

Study (Author, Year)	Study Type	Clinical Relevance (0–5)	Data Quality (0–5)	Methodological Conduct (0–5)	Robustness and Generalisability (0–5)	Reporting Quality (0–5)	Reproducibility and Transparency (0–5)	Total Score (0–30)	Key AI-Specific Concerns / Flags
K. Zhang et al., 2020 [63]	Diagnostic accuracy — internal 4-fold CV; human-in-the-loop vs LRCN & ophthalmologists	5	4	4	3	5	4	25	Single-centre; no external validation; unclear sample-size rationale; independence of model and threshold selection from the final evaluation set was insufficiently clear; sparse HP/training detail.
Yang et al., 2020 [62]	Diagnostic accuracy — internal 75/25 split; prospective trial vs ophthalmologists/parents	5	4	3	4	5	4	25	Child-level separation of training and evaluation data was not clearly reported; splitting fellow eyes across datasets could introduce information leakage; non-cycloplegic refraction as ground truth; ambiguous “external validation” phrasing; limited diversity.

Csizek et al., 2023 [61]	Diagnostic accuracy — cross-sectional; multi-site (Spain, Hungary); perceptron with repeated random splits	5	4	4	4	5	4	26	Uneven test application; incomplete application of all component tests; limited numbers of newly diagnosed cases; performance outside the two participating centres and the evaluated testing protocol remains uncertain; no public code.
Young et al., 2023 [60]	Diagnostic accuracy — prospective tele-screening (smartphone fundus); AI (ResNet-18) vs widefield & graders	5	5	5	4	5	4	28	Single-centre; small TR-ROP; pooled devices; lower specificity for RW-ROP; median-image aggregation may mask heterogeneity.
Lin et al., 2020 [59]	Diagnostic accuracy — case-control; internal 4-fold CV + external validation + prevalence simulations	5	4	5	4	5	4	27	External set referral bias; simulated prevalence ≠ real-world; parent-reported inputs; age mismatch; limited geographic diversity.
Ma et al., 2020 [58]	Diagnostic accuracy — development + preliminary field evaluation (school-based; n=100, enriched)	5	4	4	3	4	4	24	Small enriched sample; limited diversity; heuristics (fixed eyeball diameter; limbus detection); device/flash

									variability; no independent external validation, no confidence intervals.
Arnold, 2022 [57]	Diagnostic accuracy — head-to-head AI photoscreener vs established device	5	4	4	4	4	3	24	proprietary central AI system; referral-clinic spectrum bias; management of inconclusive results affects comparative performance; masking procedures were incompletely reported; author-reported conflicts of interest require consideration
Ortiz et al., 2025 [56]	Diagnostic accuracy — smartphone video; internal development with large held-out test (Mexico & Argentina)	5	5	5	4	5	4	28	Patient-level aggregation across selected frames may increase sensitivity relative to frame-level performance while reducing specificity; limited to Latin America; comparison was limited to three ophthalmologists; device/video heterogeneity; no independent out-of-site external validation.

Foo et al., 2023 [54]	Prediction model — 5-year high-myopia risk; internal CV + external school-based validation	5	5	5	4	5	5	29	Outcome imbalance (rare endpoint); school distribution shift; single-country context; calibration was not reported; confidence intervals were generated using eye-level bootstrap resampling despite inclusion of both eyes from many children, potentially underestimating uncertainty from within-child correlation.
Luo et al., 2023 [53]	Diagnostic accuracy — edge–cloud DL telemedicine (single-centre; no external validation)	5	3	3	2	3	2	18	Small and imbalanced single-centre dataset; unclear labelling procedures and inter-rater reliability; no independent external validation; no confidence intervals or calibration assessment; substantial risk of overfitting; model performance and system feasibility were not evaluated separately; no

									public code or data.
R. Zhang et al., 2023 [52]	Diagnostic accuracy - prospective validation; 5-fold subject-independent CV	5	4	5	4	5	3	26	Single-centre development and prospective validation; eye-level maximum-probability aggregation may increase apparent sensitivity; manual exclusion of low-quality images limits applicability to routine acquisition; no public code or data.

.

Table S2b. Domain-specific scoring anchors for the author-adapted six-domain rubric

Domain	0	1	2	3	4	5
**Clinical relevance**	**No identifiable clinical question or intended use**	**Clinical relationship is speculative or poorly defined**	**Potential clinical relevance, but target population, task, or intended action is unclear**	**Clearly defined clinical task and target population, but pathway relevance or intended action is incompletely described**	**Clinically important task with a clearly described population, intended use, and care-pathway relevance**	**Clearly defined, clinically consequential paediatric task with an explicit intended user, setting, comparator, decision, and downstream clinical action**
**Data quality**	**Data source, sample, and labels are not described or are fundamentally unsuitable**	**Major deficiencies in data provenance, labels, sample integrity, or reference standard**	**Substantial limitations in sample size, representativeness, missingness, labelling, or data quality**	**Data and labels are broadly adequate, but important limitations remain in representativeness, missingness, class balance, acquisition quality, or patient-level separation**	**Strong data provenance and labelling with appropriate quality control and mostly adequate sample integrity, although some representativeness or diversity limitations remain**	**Multicentre or otherwise highly representative data with rigorous reference standards, transparent quality control, appropriate sample size, patient-level separation, and explicit management of missing, poor-quality, and ungradable data**
**Methodological conduct**	**Methods are invalid or cannot support the stated conclusions**	**Major design or analytical flaws, such as clear leakage or evaluation on development data**	**Substantial methodological limitations affecting validity, including unclear data partitioning, uncontrolled tuning, or inappropriate analysis**	**Core methods are appropriate, but important limitations remain in threshold selection, clustering, missing data, validation, or uncertainty estimation**	**Rigorous model development and evaluation with clearly separated datasets and appropriate analyses, with only minor or moderate limitations**	**Fully rigorous and prespecified development and evaluation, including patient-level separation, independent testing, appropriate clustering, uncertainty estimation, threshold justification, calibration, and sensitivity analyses**
**Robustness and generalisability**	**No meaningful validation**	**Apparent performance only or resubstitution evaluation**	**Limited internal evaluation in a small, narrow, or heavily selected sample**	**Appropriate internal validation or held-out testing, but no convincing independent external or temporal validation**	**Independent external, temporal, cross-site, or cross-device evaluation, although diversity, sample size, or transportability remains limited**	**Multiple independent external and/or temporal validations across clinically relevant sites, populations, devices, operators, and subgroups, with evidence of calibration and stable performance**
**Reporting quality**	**Reporting is insufficient to**	**Major elements of population, model, reference standard,**	**Several essential methodological or**	**Core study methods and principal results are reported, but important**	**Comprehensive reporting of most study elements, analytical units,**	**Complete and transparent reporting consistent with applicable AI-specific**

	**understand the study**	**analysis, or results are missing**	**performance details are incompletely reported**	**details such as thresholds, confidence intervals, failures, calibration, or subgroup results are missing**	**performance measures, limitations, and workflow characteristics, with only minor omissions**	**guidance, including data flow, analytical units, thresholds, uncertainty, calibration, subgroup findings, failure modes, exclusions, and implementation context**
**Reproducibility and transparency**	**Reproduction is impossible from the information provided**	**Very limited model, training, preprocessing, or data documentation**	**Important details are missing and no reproducibility artefacts are available**	**Methods are sufficiently described for broad conceptual replication, but code, model artefacts, detailed parameters, or data are unavailable**	**Detailed preprocessing, architecture, training, evaluation, and version information is provided, with at least some supporting code, data, protocol, or model artefacts**	**Publicly accessible and versioned code, model specifications or weights, data or an appropriately documented accessible dataset, preprocessing pipeline, evaluation code, thresholds, and sufficient documentation for independent reproduction**

Interpretive note: Scores represent descriptive methodological and reporting characteristics, not certainty of evidence, clinical safety, or readiness for implementation. A score of 5 was reserved for unusually complete evidence within that domain and was not awarded solely because a study described a clinically important task or reported high discrimination.

Uniformly high clinical-relevance scores were expected because explicit relevance to paediatric ophthalmic care and primary-, community-, or referral-linked pathways formed part of the eligibility criteria. Consequently, this domain had limited discriminatory value across the included studies.

Table S2c: Summary of Domain and Total Scores

Measure	Clinical relevance	Data quality	Methodological conduct	Robustness and generalisability	Reporting quality	Reproducibility and transparency	Total score
Mean	5.00	4.18	4.27	3.64	4.64	3.73	25.5
Median	5	4	4	4	5	4	26
Range	5–5	3–5	3–5	2–4	3–5	2–5	18–29

Interpretation: Clinical relevance was uniformly high across all 11 studies, while reporting quality was also comparatively strong. Robustness and generalisability was the lowest-scoring domain, followed by reproducibility and transparency. The relatively high total scores were driven principally by clearly defined clinical questions, clinically relevant target conditions, and generally complete study reporting. They should not be interpreted as evidence of low risk of bias, independent external validity, adequate calibration, transportability, or readiness for routine clinical implementation. Domain-level findings were therefore interpreted alongside the design-specific risk-of-bias assessments, which were given greater weight when determining the strength of the review conclusions.

Notes on Interpretation

The author-adapted rubric was used to describe methodological and reporting characteristics across six domains. It was informed by APPRAISE-AI (Kwong et al., 2023) but does not represent an unmodified application of the original 24-item weighted instrument. Each domain was scored from 0, representing the lowest level of methodological or reporting quality, to 5, representing the highest level. The six equally weighted domain scores were summed to produce a descriptive total ranging from 0 to 30.

Total scores were not converted into categorical ratings such as Excellent, Good, Moderate, or Poor. Summed scores can conceal important weaknesses because strong clinical relevance or reporting may compensate numerically for limitations in validation, calibration, representativeness, analytical-unit selection, clustering, transparency, or reproducibility. The domain scores were therefore interpreted descriptively and alongside the design-specific risk-of-bias assessments.

FARFUM-RoP was assessed separately using the dataset-quality and applicability framework presented in Appendix 3.D. Han et al. was assessed principally using ROBINS-I (Sterne et al., 2016) because its primary objective was non-randomised treatment-effect estimation using an ML-derived counterfactual rather than development or validation of an AI clinical decision-support model.

Abbreviations: AI, artificial intelligence; CI, confidence interval; CV, cross-validation; DL, deep learning; HP, hyperparameters; IRR, inter-rater reliability; LRCN, long-term recurrent convolutional network; ML, machine learning; ROP, retinopathy of prematurity; RW-ROP, referral-warranted retinopathy of prematurity; TR-ROP, treatment-requiring retinopathy of prematurity.

Score	General interpretation
0	Domain not addressed or fundamentally inadequate
1	Major deficiencies with insufficient information
2	Substantial limitations affecting interpretability
3	Adequate conduct or reporting with important limitations
4	Strong conduct or reporting with minor or moderate limitations
5	Comprehensive, rigorous, and clearly documented evidence for the domain
