# Supplementary material for: Clinical Performance and Implementation of AI-Enabled Paediatric Ophthalmic Screening, Triage, Diagnosis, and Surveillance in Primary, Community, and Referral-Linked Pathways: A Systematic Review
Source: Diagnostics (Basel). 2026 Jul 29;16(15):2389. doi: 10.3390/diagnostics16152389 (PMC13465666; doi:10.3390/diagnostics16152389)
Supplement: Supplementary file 1 [file diagnostics-16-02389-s001.zip › S3.pdf]

Table S3a. Author-adapted QUADAS-2 risk-of-bias assessment incorporating QUADAS-AI–informed AI-specific considerations

Study (Author, Year)	Patient Selection (L/S/H)	Index Test / Model (L/S/H)	Reference Standard (L/S/H)	Flow & Timing / Data Integrity (L/S/H)	Applicability (L/S/H)	AI Transparency & Fairness (L/S/H)	Overall RoB (L/S/H)	Key AI-specific Flags / Concerns
Zhang et al. 2020 [63]	S	H	S	S	S	H	H	Incorporation bias; unclear tuning vs. evaluation separation; no external validation; missing subgroup/fairness and calibration reporting.
Yang et al. 2020 [62]	S	S	S	S	S	S	S	Noncycloplegic reference-standard limitations; possible correlated eyes; no calibration reported; limited ethnic generalizability; transfer learning domain shift from pretrained face model.
Csizek et al. 2023 [61]	S	S	L	S	S	S	S	No external validation (only internal splits). - Potential optimistic bias from weight tuning / test-version pre-selection. - Incomplete application of all tests to all subjects. - Missing calibration and fairness/subgroup analyses (inference).
Young et al. 2023 [60]	S	S	L	S	S	S	S	No external validation; small number of TR-ROP events (n=14) limiting precision; missing calibration reporting; subgroup/fairness performance not evaluated; potential

								subtle drift from combining device cohorts.
Lin et al. 2020 [59]	H	S	L	S	S	S	H	Spectrum bias (case–control), poor matching, referral bias, no calibration, absence of subgroup/fairness evaluation, and non-ideal real-world validation.
Ma et al. 2020 [58]	S	S	L	S	S	S	S	Enriched sampling (20 teacher-referred + 80 random) → spectrum bias; no external validation; heuristic threshold definitions; no calibration or subgroup/fairness reporting; narrow age/setting.
Arnold et al. 2022 [57]	S	S	L	S	S	S	S	Proprietary black-box AI; analytic choice on inconclusive handling could bias accuracy; limited fairness evaluation (beyond developmental delay); no reported calibration; spectrum/applicability enrichment.
Ortiz et al. 2025 [56]	S	S	L	S	S	S	S	Small training set relative to test set; potential optimistic bias from curated frame selection (hard negative mining); patient-level aggregation across selected frames may increase sensitivity relative to frame-level performance while reducing specificity; lack of subgroup/fairness evaluation; limited

								geographic generalizability beyond Latin America.
Luo et al. 2023 [53]	S	H	S	S	S	S	H	No external validation; single grader reference standard; unclear patient-level splitting (possible leakage); resampling strategy risks overfitting; missing calibration; no subgroup/fairness analyses; limited spectrum.
Zhang et al. 2023 [52]	S	S	S	S	S	S	S	Exclusion of low-quality images; eye-level maximum-probability aggregation may increase apparent sensitivity relative to individual-image performance; absent calibration; no fairness/subgroup reporting; single-centre validation only.

Legend: L, low risk or low concern; S, some concerns; H, high risk or high concern. The patient-selection, index-test, reference-standard, and flow-and-timing judgements were based on an author-adapted QUADAS-2 structure. AI-specific considerations, including patient-level data separation, leakage, calibration, model transparency, versioning, and subgroup fairness, were informed by the QUADAS-AI literature and incorporated as review-specific assessment criteria. Applicability and AI transparency/fairness judgments were considered separately from the overall risk-of-bias judgment.

Abbreviations: AI, artificial intelligence; RoB, risk of bias; ROP, retinopathy of prematurity; TR-ROP, treatment-requiring retinopathy of prematurity.

Table S3b. Summary PROBAST+AI assessment of the prediction-model study.

Study	Prediction target	Setting and data source	Sample size and outcome frequency	Participants and data sources (L/S/H)	Predictors (L/S/H)	Outcome (L/S/H)	Analysis (L/S/H)	Overall risk of bias (L/S/H)	Overall applicability concern (L/S/H)	Key concerns and flags
Foo et al., 2023 [54]	Development of high myopia within five years	Longitudinal Singapore school cohort; primary development/internal-evaluation data from Schools 2 and 3 and external evaluation data from School 1	Primary dataset: 965 children and 1,878 eyes; external dataset: 99 children and 189 eyes. The high-myopia outcome was uncommon and differed between datasets	S	L	L	S	S	S	School-based participants from one country; outcome imbalance; differences between development and external-evaluation schools; calibration was not reported; both eyes from many children were included; and eye-level bootstrap resampling may have underestimated uncertainty arising from within-child correlation.

Legend: L, low risk or low concern; S, some concerns; H, high risk or high concern. The assessment summarises the four PROBAST+AI domains for model performance evaluation. Applicability was considered in relation to the intended use of the model in broader paediatric school and community populations.

Abbreviations: AI, artificial intelligence; PROBAST+AI, Prediction model Risk Of Bias ASsessment Tool–Artificial Intelligence extension; RoB, risk of bias.

Legend: L = Low risk; S = Some concerns; H = High risk.
RoB = Risk of bias; PROBAST+AI = Prediction model Risk Of Bias ASsessment Tool + Artificial Intelligence extensions.

Table S3c. Risk-of-Bias Assessment Using ROBINS-I: Non-Randomised Treatment-Effect Study

Study	Design	Confounding	Selection of participants	Classification of interventions	Deviations from intended interventions	Missing data	Measurement of outcomes	Selection of the reported result	Overall risk of bias	Key concerns and flags
**Han et al., 2025 [51]**	Retrospective single-centre cohort in which outcomes among atropine-treated eyes were compared with an ML-derived natural-history counterfactual	Serious	Moderate	Low	Moderate	Moderate	Serious	Moderate	Serious	No concurrent untreated control; potential residual confounding and treatment-selection bias; adherence was not measured; both eyes from some children were analysed without adequate adjustment for within-child correlation; imperfect alignment between cycloplegic and manifest refraction measurements; and uncertainty arising from prediction error and calibration of the ML-derived counterfactual was not fully propagated into the treatment-effect estimates.

Legend: ROBINS-I judgments: Low, Moderate, Serious, Critical, or No information. ML, machine learning; ROBINS-I, Risk Of Bias In Non-randomised Studies of Interventions

Table S3d. Dataset Quality, Representativeness, and Applicability Assessment of FARFUM-RoP

Dataset	Context and intended use	Selection and representativeness (L/S/H)	Labelling and reference standard (L/S/H)	Filtering and exclusions (L/S/H)	Provenance and versioning (L/S/H)	Preprocessing transparency (L/S/H)	Annotation reliability and uncertainty (L/S/H)	Bias and fairness documentation (L/S/H)	Documentation and accessibility (L/S/H)	Overall dataset concern (L/S/H)	Key concerns and flags
**FARFUM-RoP (Akbari et al., 2024) [55]**	Public ROP image dataset intended for computer-assisted detection, research, education, and model development	S	S	S	S	S	S	S	L	S	Single-centre origin; removal of low-quality images, which reduces representation of real-world acquisition failure; image-level rather than examination-level annotations; no reported formal inter-rater reliability statistic; limited demographic and subgroup documentation; incomplete explicit version history; and no recommended patient-level development and test partitions, creating a potential risk of patient leakage in downstream studies.

Legend: L, low concern; S, some concerns; H, high concern. These judgments are descriptive assessments derived from an author-developed dataset-quality, representativeness, and applicability framework and should not be interpreted as ratings from a validated risk-of-bias instrument. FARFUM-RoP, Farabi Retina Fundus Morphology–Retinopathy of Prematurity dataset; ROP, retinopathy of prematurity.
